# Correction: A dynamic generalized fuzzy multi-criteria group decision making approach for green supplier segmentation

**DOI:** 10.1371/journal.pone.0251940

**Published:** 2021-05-13

**Authors:** Do Anh Duc, Luu Huu Van, Vincent F. Yu, Shuo-Yan Chou, Ngo Van Hien, Ngo The Chi, Dinh Van Toan, Luu Quoc Dat

In the title of this article, the word “croup” should be “group.” The correct title is: A dynamic generalized fuzzy multi-criteria group decision making approach for green supplier segmentation. The correct citation is: Duc DA, Van LH, Yu VF, Chou S-Y, Hien NV, Chi NT, et al. (2021) A dynamic generalized fuzzy multi-criteria group decision making approach for green supplier segmentation. PLoS ONE 16(1): e0245187. https://doi.org/10.1371/journal.pone.0245187
