# Supplementary material for: A dynamic generalized fuzzy multi-criteria croup decision making approach for green supplier segmentation
Source: PLoS One. 2021 Jan 25;16(1):e0245187. doi: 10.1371/journal.pone.0245187 (PMC7833157; doi:10.1371/journal.pone.0245187)
Supplement: S1 Appendix — (DOCX) [file pone.0245187.s001.docx]

**S1 Appendix**

**Appendix A**

Supplier segmentation approaches

| **Methodology** | **Segmentation method** | **Reference(s)** |
| --- | --- | --- |
| Conceptual | Process | [13] |
|  | Portfolio | [18] |
|  | Portfolio and Involvement | [37] |
| Empirical | Involvement | [29] |
|  | Portfolio and Involvement | [22-25] |
|  | AHP and Taguchi method | [21] |
|  | AHP, Fuzzy AHP | [2,8,12,13] |
|  | Fuzzy logic | [3] |
|  | Portfolio, Best Worst Method | [31,32] |
|  | Confirmatory factor analysis, VIKOR, fuzzy C-means | [11,33] |
|  | PROMETHEE, Multi-Attribute Utility Theory | [20] |

List of capabilities criteria

| **Criteria** | **Sub-criteria** | **Sub-sub-criteria or explanation** |
| --- | --- | --- |
| ***Economic criteria*** | Price/Cost | Product price, logistics cost |
|  | Quality of products | ISO quality system, repair and return rate |
|  | Delivery | Lead time, safety of components |
|  | Technology | Communication and e-commerce systems, production facilities and capacity |
|  | Flexibility | Product volume changes, using flexible machines |
|  | Financial capability | Financial position |
|  | Culture | Vendor’s image |
|  | Innovativeness | New launch of products and/or technologies |
|  | Relationship | Relationship closeness |
| ***Environ-mental criteria*** | Pollution production or control | Harmful materials released, pollution reduction capability, end-of-pipe controls |
|  | Resource consumption | Consumption of resources in terms of raw material, energy, and water |
|  | Eco-design | Design for resource efficiency, Design of products for reuse, recycle, and recovery of material |
|  | Environmental management system | Environmental certificates, environmental implementation and operation  Reverse logistics system |
|  | Green image and product | Environmental friendly product packaging, social responsibility |
|  | Green competencies | Clean technology |
|  | Staff environmental training | Staff training on environmental issues |
| ***Social criteria*** | Safety and health | Standardized health and safety conditions |
|  | Employment practices | Job stability, employee welfare |

*(Sources: [4,6,7,32])*

List of willingness criteria

| Willingness criteria | |
| --- | --- |
| Commitment to quality | Open to site evaluation |
| Commitment to continuous improvement in product and process | Prior experience with supplier |
| Commitment to greening | Impression |
| Relationship closeness | Willingness to share: information, ideas, technology, cost savings |
| Honest and frequent communication | Willingness to invest in specific technology |
| Communication richness | Willingness to co-design |
| Open to site evaluation | Willingness to participate in new product development |
| Attitude | Willingness to eliminate waste |

*(Sources: [2,3,11,12])*

**Appendix B. Preliminaries**

**Generalized fuzzy numbers**

We note that $\widetilde{H}_{1}= (\widetilde{s}_{1}, \widetilde{s}_{2}, \widetilde{s}_{3}, \widetilde{s}_{4};w_{\widetilde{H}_{1}}),0<w_{\widetilde{H}_{1}}\leq1$ is a generalized trapezoidal fuzzy number (GTrFN), where $w\in(0,1],$ $\widetilde{s}_{1},\widetilde{s}_{2},\widetilde{s}_{3}$, and $\widetilde{s}_{4}$ are real numbers. If $w_{\widetilde{H}_{1}}=1,$ then GTrFN $\widetilde{H}_{1}$ is called a normal TrFN and denoted as $\widetilde{H}_{1}= (\widetilde{s}_{1}, \widetilde{s}_{2}, \widetilde{s}_{3}, \widetilde{s}_{4};1).$ If $\widetilde{s}_{2}=\widetilde{s}_{3}$ then $\widetilde{H}_{1}$ becomes a generalized triangular fuzzy number (GTFN) and can be denoted as $\widetilde{H}_{1}= (\widetilde{s}_{1}, \widetilde{s}_{2}, \widetilde{s}_{3};w_{\widetilde{H}_{1}}).$ The membership function $\mu_{\widetilde{H}_{1}}(x)$ of GTrFN $\widetilde{H}_{1}$ satisfies the following conditions [38, 39]:

1. $\mu_{\widetilde{H}_{1}}(x)$ is continuous to $[0,w];$
2. $\mu_{\widetilde{H}_{1}}(x)=0$, for all $x\in\left(-\infty,\widetilde{s}_{1}\right];$
3. $\mu_{\widetilde{H}_{1}}(x)$ is strictly increasing in $[\widetilde{s}_{1},\widetilde{s}_{2}];$
4. $\mu_{\widetilde{H}_{1}}(x)=w,$ for all $x\in\text{[}\widetilde{s}_{2},\widetilde{s}_{3}\text{]};$
5. $\mu_{\widetilde{H}_{1}}(x)$ is strictly decreasing in $[\widetilde{s}_{3},\widetilde{s}_{4}];$
6. $\mu_{\widetilde{H}_{1}}(x)=0,$ for all $x\in\left(\widetilde{s}_{4},\infty\right]$.

**Arithmetic operations on generalized fuzzy numbers**

Let $\widetilde{H}_{1}$ and $\widetilde{H}_{2}$ be two GTrFNs; i.e., $\widetilde{H}_{1}= (\widetilde{x}_{1}, \widetilde{x}_{2}, \widetilde{x}_{3}, \widetilde{x}_{4};w_{\widetilde{H}_{1}})$ and $\widetilde{H}_{2}=(\widetilde{y}_{1},\widetilde{y}_{2},\widetilde{y}_{3},\widetilde{y}_{4};w_{\widetilde{H}_{2}}),$ where $\widetilde{x}_{1},\widetilde{x}_{2},\widetilde{x}_{3},\widetilde{x}_{4},\widetilde{y}_{1},\widetilde{y}_{2},\widetilde{y}_{3}$ and $\widetilde{y}_{4}$ are real values, $0\leq w_{\widetilde{H}_{1}}\leq1$, and $0\leq w_{\widetilde{H}_{2}}\leq1.$ Some arithmetic operators between GTrFNs $\widetilde{H}_{1}$ and $\widetilde{H}_{2}$ are defined as follows [38].

(i). Addition $(+):$

$$\begin{matrix} \widetilde{H}_{1}(+)\widetilde{H}_{2}=(\widetilde{x}_{1},\widetilde{x}_{2},\widetilde{x}_{3},\widetilde{x}_{4};w_{\widetilde{H}_{1}})(+)(\widetilde{y}_{1},\widetilde{y}_{2},\widetilde{y}_{3},\widetilde{y}_{4};w_{\widetilde{H}_{2}}) \\ =(\widetilde{x}_{1}+\widetilde{y}_{1},\widetilde{x}_{2}+\widetilde{y}_{2},\widetilde{x}_{3}+\widetilde{y}_{3},\widetilde{x}_{4}+\widetilde{y}_{4};\min(w_{\widetilde{H}_{1}},w_{\widetilde{H}_{2}})) \end{matrix}$$

(ii). Subtraction $(-):$

$$\begin{matrix} \widetilde{H}_{1}(-)\widetilde{H}_{2}=(\widetilde{x}_{1},\widetilde{x}_{2},\widetilde{x}_{3},\widetilde{x}_{4};w_{\widetilde{H}_{1}})(-)(\widetilde{y}_{1},\widetilde{y}_{2},\widetilde{y}_{3},\widetilde{y}_{4};w_{\widetilde{H}_{2}}) \\ =(\widetilde{x}_{1}-\widetilde{y}_{4},\widetilde{x}_{2}-\widetilde{y}_{3},\widetilde{x}_{3}-\widetilde{y}_{2},\widetilde{x}_{4}-\widetilde{y}_{1};\min(w_{\widetilde{H}_{1}},w_{\widetilde{H}_{2}})) \end{matrix}$$

(iii). Multiplication $(\text{x}):$

$$\begin{matrix} \widetilde{H}_{1}(\text{x})\widetilde{H}_{2}=(\widetilde{x}_{1},\widetilde{x}_{2},\widetilde{x}_{3},\widetilde{x}_{4};w_{\widetilde{H}_{1}})(\mathrm{x})(\widetilde{y}_{1},\widetilde{y}_{2},\widetilde{y}_{3},\widetilde{y}_{4};w_{\widetilde{H}_{2}}) \\ =(\widetilde{x}_{1}\times\widetilde{y}_{1},\widetilde{x}_{2}\times\widetilde{y}_{2},\widetilde{x}_{3}\times\widetilde{y}_{3},\widetilde{x}_{4}\times\widetilde{y}_{4};\min(w_{\widetilde{H}_{1}},w_{\widetilde{H}_{2}})) \end{matrix}$$

(iv). Division

$$\begin{matrix} \widetilde{H}_{1}(/)\widetilde{H}_{2}=(\widetilde{x}_{1},\widetilde{x}_{2},\widetilde{x}_{3},\widetilde{x}_{4};w_{\widetilde{H}_{1}})(/)(\widetilde{y}_{1},\widetilde{y}_{2},\widetilde{y}_{3},\widetilde{y}_{4};w_{\widetilde{H}_{2}}) \\ =(\widetilde{x}_{1}/\widetilde{y}_{4},\widetilde{x}_{2}/\widetilde{y}_{3},\widetilde{x}_{3}/\widetilde{y}_{2},\widetilde{x}_{4}/\widetilde{y}_{1};\min(w_{\widetilde{H}_{1}},w_{\widetilde{H}_{2}})) \end{matrix}$$

Here, $\widetilde{x}_{1},\widetilde{x}_{2},\widetilde{x}_{3},\widetilde{x}_{4},\widetilde{y}_{1},\widetilde{y}_{2},\widetilde{y}_{3}$ and $\widetilde{y}_{4}$ are non-zero positive real numbers.

**Linguistic variables and fuzzy numbers**

Table 1 shows the linguistic variables represented by GTFNs for the ratings of alternatives and the importance weights of the criteria [40].

**Table 1.** Ratings of alternatives and importance weights of the criteria

| **Ratings** | | **Importance weights** | |
| --- | --- | --- | --- |
| Linguistic variable | GTFNs | Linguistic variable | GTFNs |
| Very Poor (VP) | (0.1, 0.2, 0.3; 0.6) | Unimportant (UI) | (0.0, 0.2, 0.4; 0.6) |
| Poor (P) | (0.2, 0.3, 0.4; 0.7) | Ordinary Important (OI) | (0.3, 0.4, 0.5; 0.7) |
| Fair (F) | (0.3, 0.5, 0.7; 0.8) | Important (I) | (0.4, 0.5, 0.6; 0.8) |
| Good (G) | (0.5, 0.7, 0.9; 0.9) | Very Important (VI) | (0.5, 0.7, 0.9; 0.9) |
| Very Good (VG) | (0.8, 0.9, 1.0; 1.0) | Absolutely Important (AI) | (0.8, 0.9, 1.0; 0.9) |

**Appendix C**

**Table 2a.** Average ratings of suppliers versus the capabilities criteria

| **Criterion** | **Supplier** | **Decision maker** | | | | | | | | | **Aggregated ratings** *r*_ij_ |
| --- | --- | --- | --- | --- | --- | --- | --- | --- | --- | --- | --- |
|  |  | *t*_1_ | | | *t*_2_ | | | *t*_3_ | | |  |
|  |  | *D*_1_ | *D*_2_ | *D*_3_ | *D*_1_ | *D*_2_ | *D*_3_ | *D*_1_ | *D*_2_ | *D*_3_ |  |
| *C*_1_ | *A*_1_ | F | F | F | P | F | P | P | F | P | (0,256, 0,411, 0,567; 0,700) |
|  | *A*_2_ | F | P | F | F | F | F | P | F | F | (0,278, 0,456, 0,633; 0,700) |
|  | *A*_3_ | VG | G | G | VG | G | G | G | VG | G | (0,600, 0,767, 0,933; 0,900) |
|  | *A*_4_ | VP | P | VP | P | P | VP | P | F | P | (0,178, 0,289, 0,400; 0,600) |
|  | *A*_5_ | G | G | G | G | G | F | VG | G | G | (0,511, 0,700, 0,889; 0,800) |
|  | *A*_6_ | G | F | F | G | G | G | F | F | F | (0,389, 0,589, 0,789; 0,800) |
|  | *A*_7_ | VG | VG | VG | G | VG | VG | VG | G | VG | (0,733, 0,856, 0,978; 0,900) |
|  | *A*_8_ | VP | VP | VP | P | VP | P | P | F | F | (0,178, 0,300, 0,422; 0,600) |
|  | *A*_9_ | G | F | G | G | G | G | G | G | G | (0,478, 0,678, 0,878; 0,800) |
|  | *A*_10_ | VG | G | G | VG | G | VG | G | VG | G | (0,633, 0,789, 0,944; 0,900) |
|  | *A*_11_ | F | G | F | F | G | F | F | F | F | (0,344, 0,544, 0,744; 0,800) |
|  | *A*_12_ | P | P | F | F | P | F | F | P | F | (0,256, 0,411, 0,567; 0,700) |
| *C*_2_ | *A*_1_ | G | F | G | G | G | G | F | F | G | (0,433, 0,633, 0,833; 0,800) |
|  | *A*_2_ | P | VP | VP | P | F | F | P | F | F | (0,222, 0,367, 0,511; 0,600) |
|  | *A*_3_ | G | G | VG | VG | G | VG | VG | G | G | (0,633, 0,789, 0,944; 0,900) |
|  | *A*_4_ | F | P | F | F | F | F | P | F | F | (0,278, 0,456, 0,633; 0,700) |
|  | *A*_5_ | F | F | P | F | F | F | F | F | G | (0,311, 0,500, 0,689; 0,700) |
|  | *A*_6_ | G | G | G | VG | G | G | VG | G | VG | (0,600, 0,767, 0,933; 0,900) |
|  | *A*_7_ | VG | G | VG | G | G | VG | G | G | VG | (0,633, 0,789, 0,944; 0,900) |
|  | *A*_8_ | P | F | P | P | F | P | F | G | G | (0,300, 0,456, 0,611; 0,700) |
|  | *A*_9_ | F | F | F | F | F | F | P | F | F | (0,289, 0,478, 0,667; 0,700) |
|  | *A*_10_ | P | VP | P | P | F | F | P | F | F | (0,233, 0,378, 0,522; 0,600) |
|  | *A*_11_ | G | VG | G | G | VG | G | G | G | VG | (0,600, 0,767, 0,933; 0,900) |
|  | *A*_12_ | G | G | F | P | VP | P | F | P | F | (0,289, 0,444, 0,600; 0,600) |

**Table 2b.** Average ratings of suppliers versus the capabilities criteria

| **Criterion** | **Supplier** | **Decision maker** | | | | | | | | | **Aggregated ratings** *r*_ij_ |
| --- | --- | --- | --- | --- | --- | --- | --- | --- | --- | --- | --- |
|  |  | *t*_1_ | | | *t*_2_ | | | *t*_3_ | | |  |
|  |  | *D*_1_ | *D*_2_ | *D*_3_ | *D*_1_ | *D*_2_ | *D*_3_ | *D*_1_ | *D*_2_ | *D*_3_ |  |
| *C*_3_ | *A*_1_ | F | F | G | G | F | G | F | F | G | (0,389, 0,589, 0,789; 0,800) |
|  | *A*_2_ | P | VP | P | F | F | P | P | VP | P | (0,200, 0,322, 0,444; 0,600) |
|  | *A*_3_ | G | VG | VG | G | G | G | VG | G | G | (0,600, 0,767, 0,933; 0,900) |
|  | *A*_4_ | F | F | F | P | F | P | P | F | P | (0,256, 0,411, 0,567; 0,700) |
|  | *A*_5_ | F | F | G | VG | VG | G | VG | G | G | (0,556, 0,722, 0,889; 0,800) |
|  | *A*_6_ | G | F | G | G | G | G | G | F | G | (0,456, 0,656, 0,856; 0,800) |
|  | *A*_7_ | F | F | P | F | VG | VG | G | F | G | (0,444, 0,611, 0,778; 0,700) |
|  | *A*_8_ | F | F | F | F | F | G | F | G | F | (0,344, 0,544, 0,744; 0,800) |
|  | *A*_9_ | G | F | G | G | F | F | G | VG | G | (0,467, 0,656, 0,844; 0,800) |
|  | *A*_10_ | F | F | F | F | F | F | G | G | VG | (0,400, 0,589, 0,778; 0,800) |
|  | *A*_11_ | G | G | F | G | G | VG | G | F | F | (0,467, 0,656, 0,844; 0,800) |
|  | *A*_12_ | F | F | P | P | F | F | F | P | F | (0,267, 0,433, 0,600; 0,700) |
| *C*_4_ | *A*_1_ | G | F | G | G | VG | G | VG | G | G | (0,544, 0,722, 0,900; 0,800) |
|  | *A*_2_ | F | P | F | F | P | F | F | P | F | (0,267, 0,433, 0,600; 0,700) |
|  | *A*_3_ | G | VG | G | VG | VG | G | G | G | G | (0,600, 0,767, 0,933; 0,900) |
|  | *A*_4_ | G | F | G | P | VP | P | F | F | P | (0,289, 0,444, 0,600; 0,600) |
|  | *A*_5_ | F | G | G | F | F | G | G | G | G | (0,433, 0,633, 0,833; 0,800) |
|  | *A*_6_ | F | P | F | F | G | F | F | G | F | (0,333, 0,522, 0,711; 0,700) |
|  | *A*_7_ | VG | VG | G | G | VG | VG | VG | G | G | (0,667, 0,811, 0,956; 0,900) |
|  | *A*_8_ | P | F | P | P | F | P | F | G | F | (0,278, 0,433, 0,589; 0,700) |
|  | *A*_9_ | F | F | F | G | F | F | G | VG | G | (0,422, 0,611, 0,800; 0,800) |
|  | *A*_10_ | G | G | VG | G | F | G | VG | G | G | (0,544, 0,722, 0,900; 0,800) |
|  | *A*_11_ | G | F | F | G | F | F | G | G | VG | (0,444, 0,633, 0,822; 0,800) |
|  | *A*_12_ | P | VP | P | F | F | P | F | F | P | (0,233, 0,378, 0,522; 0,600) |

**Table 2c.** Average ratings of suppliers versus the capabilities criteria

| **Criterion** | **Supplier** | **Decision maker** | | | | | | | | | **Aggregated ratings** *r*_ij_ |
| --- | --- | --- | --- | --- | --- | --- | --- | --- | --- | --- | --- |
|  |  | *t*_1_ | | | *t*_2_ | | | *t*_3_ | | |  |
|  |  | *D*_1_ | *D*_2_ | *D*_3_ | *D*_1_ | *D*_2_ | *D*_3_ | *D*_1_ | *D*_2_ | *D*_3_ |  |
| *C*_5_ | *A*_1_ | G | G | F | G | VG | G | F | G | G | (0,489, 0,678, 0,867; 0,800) |
|  | *A*_2_ | F | P | F | P | F | P | P | VP | P | (0,222, 0,356, 0,489; 0,600) |
|  | *A*_3_ | G | VG | G | VG | G | G | F | F | G | (0,522, 0,700, 0,878; 0,800) |
|  | *A*_4_ | G | G | G | VP | P | P | P | P | F | (0,300, 0,444, 0,589; 0,600) |
|  | *A*_5_ | P | F | F | F | F | F | G | F | G | (0,333, 0,522, 0,711; 0,700) |
|  | *A*_6_ | G | G | F | F | G | F | G | G | VG | (0,467, 0,656, 0,844; 0,800) |
|  | *A*_7_ | VG | VG | VG | G | VG | G | G | G | VG | (0,667, 0,811, 0,956; 0,900) |
|  | *A*_8_ | VP | P | VP | F | P | F | F | F | F | (0,233, 0,389, 0,544; 0,600) |
|  | *A*_9_ | G | F | G | VG | G | G | VG | G | G | (0,544, 0,722, 0,900; 0,800) |
|  | *A*_10_ | G | F | G | G | VG | G | G | G | G | (0,511, 0,700, 0,889; 0,800) |
|  | *A*_11_ | G | G | G | G | G | G | G | F | G | (0,478, 0,678, 0,878; 0,800) |
|  | *A*_12_ | F | P | P | F | F | P | F | F | P | (0,256, 0,411, 0,567; 0,700) |
| *C*_6_ | *A*_1_ | F | F | G | G | F | G | G | G | G | (0,433, 0,633, 0,833; 0,800) |
|  | *A*_2_ | F | P | P | F | P | F | F | P | P | (0,244, 0,389, 0,533; 0,700) |
|  | *A*_3_ | G | G | VG | F | G | G | VG | G | VG | (0,578, 0,744, 0,911; 0,800) |
|  | *A*_4_ | P | F | F | P | F | P | F | P | P | (0,244, 0,389, 0,533; 0,700) |
|  | *A*_5_ | G | F | G | F | F | G | G | VG | G | (0,467, 0,656, 0,844; 0,800) |
|  | *A*_6_ | VG | G | G | G | F | G | G | F | G | (0,489, 0,678, 0,867; 0,800) |
|  | *A*_7_ | P | P | P | F | P | P | G | G | F | (0,289, 0,433, 0,578; 0,700) |
|  | *A*_8_ | F | F | F | P | F | F | F | P | F | (0,278, 0,456, 0,633; 0,700) |
|  | *A*_9_ | G | G | VG | G | F | G | G | F | F | (0,467, 0,656, 0,844; 0,800) |
|  | *A*_10_ | G | VG | G | G | VG | G | VG | VG | G | (0,633, 0,789, 0,944; 0,900) |
|  | *A*_11_ | G | F | G | VG | G | G | G | G | G | (0,511, 0,700, 0,889; 0,800) |
|  | *A*_12_ | VP | P | VP | F | P | F | F | F | P | (0,222, 0,367, 0,511; 0,600) |

**Table 2d.** Average ratings of suppliers versus the willingness criteria

| **Criterion** | **Supplier** | **Decision maker** | | | | | | | | | **Aggregated ratings** *r*_ij_ |
| --- | --- | --- | --- | --- | --- | --- | --- | --- | --- | --- | --- |
|  |  | *t*_1_ | | | *t*_2_ | | | *t*_3_ | | |  |
|  |  | *D*_1_ | *D*_2_ | *D*_3_ | *D*_1_ | *D*_2_ | *D*_3_ | *D*_1_ | *D*_2_ | *D*_3_ |  |
| *W*_1_ | *A*_1_ | P | F | P | F | F | P | G | F | G | (0,311, 0,478, 0,644; 0,700) |
|  | *A*_2_ | G | VG | VG | G | G | G | G | G | VG | (0,600, 0,767, 0,933; 0,900) |
|  | *A*_3_ | G | F | G | VG | G | G | VG | G | G | (0,544, 0,722, 0,900; 0,800) |
|  | *A*_4_ | VG | G | G | G | VG | G | G | G | G | (0,567, 0,744, 0,922; 0,900) |
|  | *A*_5_ | G | F | G | G | G | G | F | F | G | (0,433, 0,633, 0,833; 0,800) |
|  | *A*_6_ | G | VG | G | G | G | G | VG | G | G | (0,567, 0,744, 0,922; 0,900) |
|  | *A*_7_ | G | G | G | F | G | G | G | F | G | (0,456, 0,656, 0,856; 0,800) |
|  | *A*_8_ | P | F | F | P | F | P | F | G | F | (0,289, 0,456, 0,622; 0,700) |
|  | *A*_9_ | G | G | VG | G | F | G | F | G | G | (0,489, 0,678, 0,867; 0,800) |
|  | *A*_10_ | F | P | F | G | F | G | G | F | F | (0,356, 0,544, 0,733; 0,700) |
|  | *A*_11_ | F | G | F | G | G | F | G | F | G | (0,411, 0,611, 0,811; 0,800) |
|  | *A*_12_ | P | VP | P | P | P | F | G | VG | G | (0,333, 0,467, 0,600; 0,600) |
| *W*_2_ | *A*_1_ | F | P | F | F | P | F | F | P | F | (0,267, 0,433, 0,600; 0,700) |
|  | *A*_2_ | G | G | VG | F | G | F | G | G | VG | (0,522, 0,700, 0,878; 0,800) |
|  | *A*_3_ | G | F | G | G | G | G | G | G | G | (0,478, 0,678, 0,878; 0,800) |
|  | *A*_4_ | F | G | G | G | F | G | G | F | G | (0,433, 0,633, 0,833; 0,800) |
|  | *A*_5_ | F | G | G | G | VG | G | VG | G | G | (0,544, 0,722, 0,900; 0,800) |
|  | *A*_6_ | F | F | G | G | G | VG | G | F | G | (0,467, 0,656, 0,844; 0,800) |
|  | *A*_7_ | G | G | VG | G | G | G | G | G | G | (0,533, 0,722, 0,911; 0,900) |
|  | *A*_8_ | P | VP | P | P | P | F | F | F | G | (0,256, 0,400, 0,544; 0,600) |
|  | *A*_9_ | G | G | F | G | F | F | F | G | F | (0,389, 0,589, 0,789; 0,800) |
|  | *A*_10_ | G | F | F | G | G | F | G | F | G | (0,411, 0,611, 0,811; 0,800) |
|  | *A*_11_ | F | G | G | G | G | G | G | G | VG | (0,511, 0,700, 0,889; 0,800) |
|  | *A*_12_ | G | G | G | G | F | G | G | F | G | (0,456, 0,656, 0,856; 0,800) |

**Table 2e.** Average ratings of suppliers versus the willingness criteria

| **Criterion** | **Supplier** | **Decision maker** | | | | | | | | | **Aggregated ratings *r*_ij_** |
| --- | --- | --- | --- | --- | --- | --- | --- | --- | --- | --- | --- |
|  |  | *t*_1_ | | | *t*_2_ | | | *t*_3_ | | |  |
|  |  | *D*_1_ | *D*_2_ | *D*_3_ | *D*_1_ | *D*_2_ | *D*_3_ | *D*_1_ | *D*_2_ | *D*_3_ |  |
| *W*_3_ | *A*_1_ | P | P | P | F | P | F | F | P | F | (0,244, 0,389, 0,533; 0,700) |
|  | *A*_2_ | F | F | G | F | G | F | G | G | G | (0,411, 0,611, 0,811; 0,800) |
|  | *A*_3_ | G | G | F | F | G | F | F | G | F | (0,389, 0,589, 0,789; 0,800) |
|  | *A*_4_ | F | G | F | G | G | G | F | G | G | (0,433, 0,633, 0,833; 0,800) |
|  | *A*_5_ | G | F | G | G | F | G | G | G | G | (0,456, 0,656, 0,856; 0,800) |
|  | *A*_6_ | F | G | F | G | F | F | VG | G | VG | (0,478, 0,656, 0,833; 0,800) |
|  | *A*_7_ | G | VG | VG | G | VG | G | G | G | G | (0,600, 0,767, 0,933; 0,900) |
|  | *A*_8_ | P | P | P | F | P | F | F | F | G | (0,278, 0,433, 0,589; 0,700) |
|  | *A*_9_ | G | VG | G | VG | VG | G | F | G | G | (0,578, 0,744, 0,911; 0,800) |
|  | *A*_10_ | F | G | G | G | G | G | G | VG | G | (0,511, 0,700, 0,889; 0,800) |
|  | *A*_11_ | G | G | G | G | VG | G | G | G | G | (0,533, 0,722, 0,911; 0,900) |
|  | *A*_12_ | VG | G | G | G | G | G | G | G | VG | (0,567, 0,744, 0,922; 0,900) |
| *W*_4_ | *A*_1_ | F | F | F | P | F | F | G | F | G | (0,333, 0,522, 0,711; 0,700) |
|  | *A*_2_ | G | G | G | G | F | G | F | G | F | (0,433, 0,633, 0,833; 0,800) |
|  | *A*_3_ | F | G | F | G | G | F | G | G | F | (0,411, 0,611, 0,811; 0,800) |
|  | *A*_4_ | G | VG | G | G | G | G | G | G | G | (0,533, 0,722, 0,911; 0,900) |
|  | *A*_5_ | F | F | F | P | F | P | G | F | G | (0,322, 0,500, 0,678; 0,700) |
|  | *A*_6_ | F | G | G | F | F | G | VG | VG | G | (0,500, 0,678, 0,856; 0,800) |
|  | *A*_7_ | F | F | G | G | F | F | G | F | F | (0,367, 0,567, 0,767; 0,800) |
|  | *A*_8_ | F | F | F | F | G | F | G | F | G | (0,367, 0,567, 0,767; 0,800) |
|  | *A*_9_ | G | G | G | F | G | F | G | F | G | (0,433, 0,633, 0,833; 0,800) |
|  | *A*_10_ | F | G | F | F | G | G | G | G | F | (0,411, 0,611, 0,811; 0,800) |
|  | *A*_11_ | G | F | G | F | F | F | G | G | G | (0,411, 0,611, 0,811; 0,800) |
|  | *A*_12_ | G | G | G | G | VG | G | VG | G | VG | (0,600, 0,767, 0,933; 0,900) |

**References**

37. Masella C, Rangone A. A contingent approach to the design of vendor selection systems for different types of co-operative customer/supplier relationships. Int J Oper Prod. Manage 2000;20;70-84. https://doi.org/10.1108/01443570010287044

38. Chen SH. Ranking fuzzy numbers with maximizing set and minimizing set. Fuzzy Sets Syst 1985;17;113-129. https://doi.org/10.1016/0165-0114(85)90050-8

39. Hsieh CH, Chen SH. Similarity of generalized fuzzy numbers with graded mean integration representation. Proc 8th International fuzzy System Association World Congress, Taipei, Taiwan, Republic of China 1999;2;551-555.

40. Zimmermann HJ. Fuzzy Set Theory and its Applications. Kluwer Academic Publishers: Boston; 1991.
