# Supplementary material for: A dynamic generalized fuzzy multi-criteria croup decision making approach for green supplier segmentation
Source: PLoS One. 2021 Jan 25;16(1):e0245187. doi: 10.1371/journal.pone.0245187 (PMC7833157; doi:10.1371/journal.pone.0245187)
Supplement: Attachment — Submitted filename: PONE-D-20-03268_R1_reviewer.pdf [file pone.0245187.s003.pdf]

PLOS ONE

A DYNAMIC GENERALIZED FUZZY MULTI-CRITERIA GROUP DECISION MAKING APPROACH FOR GREEN SUPPLIER SEGMENTATION

--Manuscript Draft--

**Manuscript Number:**	PONE-D-20-03268R1
**Article Type:**	Research Article
**Full Title:**	A DYNAMIC GENERALIZED FUZZY MULTI-CRITERIA GROUP DECISION MAKING APPROACH FOR GREEN SUPPLIER SEGMENTATION
**Short Title:**	FUZZY MULTI-CRITERIA GROUP DECISION MAKING APPROACH FOR GREEN SUPPLIER SEGMENTATION
**Corresponding Author:**	Dat Luu Quoc, Ph.D<br>Vietnam National University<br>Hanoi, VIET NAM
**Keywords:**	Supplier segmentation; dynamic fuzzy MCGDM; centroid-index; generalized fuzzy numbers
**Abstract:**	Supplier selection and segmentation are crucial tasks of companies in order to reduce costs and increase the competitiveness of their goods. To handle uncertainty and dynamicity in the supplier segmentation problem, this research thus proposes a new dynamic generalized fuzzy multi-criteria group decision making (MCGDM) approach from the aspects of capability and willingness and with respect to environmental issues. The proposed approach defines the aggregated ratings of alternatives, the aggregated weights of criteria, and the weighted ratings by using generalized fuzzy numbers with the effect of time weight. Next, we determine the ranking order of alternatives via a popular centroid-index ranking approach. Finally, two case studies demonstrate the efficiency of the proposed dynamic approach. Then where is the result?? You only put the procedures of the work
**Order of Authors:**	Van Luu Huu
	Duc Do Anh
	Yu Vincent F.
	Chou Shuo-Yan
	Hien Ngo Van
	Chi Ngo The
	Toan Dinh Van
	Dat Luu Quoc, Ph.D
**Response to Reviewers:**	Response to Referees' Comments<br>PONE-D-20-03268<br>A DYNAMIC GENERALIZED FUZZY MULTI-CRITERIA GROUP DECISION MAKING APPROACH FOR GREEN SUPPLIER SEGMENTATION<br>The authors greatly appreciate the time and effort the referees spent on reviewing this manuscript. This paper has been revised based on the constructive comments and suggestions made by the referees. Major changes are shown in red color.<br>Referee 1's Comments:<br>I would like to thank the authors for this interesting approach in dealing with an important subject. The subject of segmentation and especially weighting using decision makers or "experts" are one of the areas of debate in many fields. I found this approach easy to follow and reproducible. This is an important advantage for the proposed method. However, I have few issues that I want to recommend and clarify:<br>1.I am not sure that you have presented enough discussion in the literature about the added value for your approach and the need for this approach, not only from operational point of view but also from computational one. In other words, you provided in the second paragraph of the introduction why you are proposing this approach, but there is no justification for where you think it will improve the current method statistically. Is this approach better in your opinion and why?

2.Tables 6a - 6e are a bit confusing within the manuscript, maybe including these tables in an annex will be more convenient.
3.There must be a discussion section after the section "Comparison of the proposed method with another fuzzy MCDM method" and the conclusion section. The results are not discussed clearly for the reader. The results need to be interpreted from mathematical and operational point of view, as a reader I am afraid I need more explanation for the numbers. It looked in some places that you jumped from section to another without explaining the results. Moreover, you need to discuss the strengths of your approach, how it tackled current existing problem, and why do you think it should be considered by others. For example, have you considered a simulation study and compare the results with other methods to assess the consistency of the results?! or have you considered comparing this method with more statistically based approaches such as Multidimensional Latent Class Item Response Theory Models?! There should be more discussion before you present your conclusion.
Responses:
1.Thank you very much for your comments. The authors have added some sentences in the introduction and literature review section to discuss more about the shortcomings of the existing approaches and the advantages of our approach.
2.Thanks for your suggestion. The authors have moved Tables 6a - 6e to appendix section.
3.Thanks for your comments. The authors have added some paragraphs to discuss about the results of the study and the advantages of our approach. Some sentences have been added in the implementation section to explain more about the calculation process. In this study, a new dynamic generalized fuzzy MCDM approach has been proposed. Then, we have compared the proposed method with another fuzzy MCDM method to show its advantages. The comparison between our proposed approach with more statistically based approaches such as Multidimensional Latent Class Item Response Theory Models should be our further research.
Referee 2’s Comments:
While new methods for "green" supplier segmentation is certainly important, interesting, and relevant, there are several issues in this paper.
1.The methods in this paper appear to be sound, it is very hard to read and comprehend. The organization and visualization of data/results is overall, poor.
2.Background on fuzzy numbers is lengthy and a bit hard to follow.
3.There is an excess of tables, which is incredibly overwhelming and unhelpful given the complexity of the topic and notation. The tables in the literature review section are redundant or unnecessary. If tables really are necessary, for this many tables, they belong in an appendix.
4.Some terms or abbreviations are not explained and confusing. For example, in Table 6a, I'm assuming "fa" = "fair", "Ve_go" = "Very good"? This needs to be standardized and presented in a more meaningful, insightful, and visually interesting manner. For example, map responses to numbers rather than letter abbreviations, and plot a heat map of responses, rather than use a table. This can be done with ALL of the tables in this section.
5.Table 8 may be better off as some sort of visual representation (chart) rather than a table.
6.There are grammatical mistakes throughout the paper
Responses:
1.Thank you very much for your comments. The authors have added some sentences in the implementation section to explain more about the data and results of this study. The authors have also moved the Tables 6a - 6e to the appendix.
2.Thanks for your suggestion. The authors have moved the background on fuzzy numbers to appendix.
3.Thanks for your suggestion. The authors have moved Tables 1-3 to the appendix.
4.Thanks for your suggestion. The authors have tried to change the abbreviations of linguistic variables (Appendix B - Table 2 and other tables).
5.Thanks for your suggestion. The authors have modified the Table 8 to make it more visually.
6.The authors have tried to fix the grammatical mistakes throughout the paper.

The authors would like to thank again the reviewers for the time and expertise they have invested in these reviews. The revised manuscript with marked changes has been resubmitted to your journal. We look forward to your positive response.

Powered by Editorial Manager® and ProduXion Manager® from Aries Systems Corporation

	Sincerely,<br>Luu Quoc Dat
Additional Information:	
Question	Response
**Financial Disclosure**<br><br>Enter a financial disclosure statement that describes the sources of funding for the work included in this submission. Review the submission guidelines for detailed requirements. View published research articles from *PLOS ONE* for specific examples.<br><br>This statement is required for submission and **will appear in the published article** if the submission is accepted. Please make sure it is accurate.<br><br>**Unfunded studies**<br>Enter: *The author(s) received no specific funding for this work.*<br><br>**Funded studies**<br>Enter a statement with the following details:<br>• Initials of the authors who received each award<br>• Grant numbers awarded to each author<br>• The full name of each funder<br>• URL of each funder website<br>• Did the sponsors or funders play any role in the study design, data collection and analysis, decision to publish, or preparation of the manuscript?<br>• **NO** - Include this sentence at the end of your statement: *The funders had no role in study design, data collection and analysis, decision to publish, or preparation of the manuscript.*<br>• **YES** - Specify the role(s) played.<br><br>* typeset	This research is funded by “VNU University of Economics and Business, Vietnam National University, Hanoi” and “Korea Foundation for Advanced Studies (KFAS) and the Asia Research Center, Vietnam National University, Hanoi (ARC-VNU)” under project number CA.18.2A. This research was completed during and after the stay of the seventh author at the Vietnam Institute for Advanced Study in Mathematics (VIASM).
**Competing Interests**<br><br>Use the instructions below to enter a competing interest statement for this submission. On behalf of all authors, disclose any competing interests that could be perceived to bias this work—acknowledging all financial support and any other relevant financial or non-	The authors have declared that no competing interests exist.

Powered by Editorial Manager® and ProduXion Manager® from Aries Systems Corporation

financial competing interests.<br><br>This statement **will appear in the published article** if the submission is accepted. Please make sure it is accurate. View published research articles from *PLOS ONE* for specific examples.<br><br>**NO authors have competing interests**<br><br>Enter: *The authors have declared that no competing interests exist.*<br><br>**Authors with competing interests**<br><br>Enter competing interest details beginning with this statement:<br><br>*I have read the journal's policy and the authors of this manuscript have the following competing interests: [insert competing interests here]*<br><br>* typeset	
**Ethics Statement**<br><br>Enter an ethics statement for this submission. This statement is required if the study involved:<br><br>• Human participants<br>• Human specimens or tissue<br>• Vertebrate animals or cephalopods<br>• Vertebrate embryos or tissues<br>• Field research<br><br>Write "N/A" if the submission does not require an ethics statement.<br><br>General guidance is provided below. Consult the submission guidelines for detailed instructions. **Make sure that all information entered here is included in the Methods section of the manuscript.**	N/A

**Format for specific study types**<br><br>**Human Subject Research (involving human participants and/or tissue)**<br>• Give the name of the institutional review board or ethics committee that approved the study<br>• Include the approval number and/or a statement indicating approval of this research<br>• Indicate the form of consent obtained (written/oral) or the reason that consent was not obtained (e.g. the data were analyzed anonymously)<br><br>**Animal Research (involving vertebrate animals, embryos or tissues)**<br>• Provide the name of the Institutional Animal Care and Use Committee (IACUC) or other relevant ethics board that reviewed the study protocol, and indicate whether they approved this research or granted a formal waiver of ethical approval<br>• Include an approval number if one was obtained<br>• If the study involved *non-human primates*, add *additional details* about animal welfare and steps taken to ameliorate suffering<br>• If anesthesia, euthanasia, or any kind of animal sacrifice is part of the study, include briefly which substances and/or methods were applied<br><br>**Field Research**<br><br>Include the following details if this study involves the collection of plant, animal, or other materials from a natural setting:<br>• Field permit number<br>• Name of the institution or relevant body that granted permission	
**Data Availability**<br><br>Authors are required to make all data underlying the findings described fully available, without restriction, and from the time of publication. PLOS allows rare exceptions to address legal and ethical concerns. See the PLOS Data Policy and FAQ for detailed information.	Yes - all data are fully available without restriction

Powered by Editorial Manager® and ProduXion Manager® from Aries Systems Corporation

A Data Availability Statement describing where the data can be found is required at submission. Your answers to this question constitute the Data Availability Statement and **will be published in the article**, if accepted.<br><br>**Important:** Stating 'data available on request from the author' is not sufficient. If your data are only available upon request, select 'No' for the first question and explain your exceptional situation in the text box.<br><br>Do the authors confirm that all data underlying the findings described in their manuscript are fully available without restriction?	
**Describe where the data may be found in full sentences. If you are copying our sample text, replace any instances of XXX with the appropriate details.**<br><br>• If the data are **held or will be held in a public repository**, include URLs, accession numbers or DOIs. If this information will only be available after acceptance, indicate this by ticking the box below. For example: *All XXX files are available from the XXX database (accession number(s) XXX, XXX.)*.<br>• If the data are all contained **within the manuscript and/or Supporting Information files**, enter the following: *All relevant data are within the manuscript and its Supporting Information files.*<br>• If neither of these applies but you are able to provide **details of access elsewhere**, with or without limitations, please do so. For example:<br><br>*Data cannot be shared publicly because of [XXX]. Data are available from the XXX Institutional Data Access / Ethics Committee (contact via XXX) for researchers who meet the criteria for access to confidential data.*<br><br>*The data underlying the results presented in the study are available from (include the name of the third party*	All relevant data are within the manuscript and its Supporting Information files.

Powered by Editorial Manager® and ProduXion Manager® from Aries Systems Corporation

and contact information or URL).<br>• This text is appropriate if the data are owned by a third party and authors do not have permission to share the data.<br><br>* typeset	
Additional data availability information:	

Powered by Editorial Manager® and ProduXion Manager® from Aries Systems Corporation

Manuscript Click here to access/download;Manuscript;Revised Manuscript R1.docx

A DYNAMIC GENERALIZED FUZZY MULTI-CRITERIA GROUP DECISION MAKING APPROACH FOR GREEN SUPPLIER SEGMENTATION

Luu Huu Van[b], Do Anh Duc[a,b], Vincent F. Yu[c], Shuo-Yan Chou[c], Ngo Van Hien[d], Ngo The Chi[e], Dinh Van Toan[b], Luu Quoc Dat[b,*]

[a] *National Economics University, 207 Giai Phong Road, Hanoi*
[b] *VNU University of Economics and Business, Vietnam National University, Hanoi 144 Xuan Thuy Road, Hanoi, Vietnam*
[c] *Department of Industrial Management, National Taiwan University of Science and Technology, 43, Section 4, Keelung Road, Taipei 10607, Taiwan*
[d] *Phenikaa University, Yen Nghia Ward, Ha Dong District, Hanoi, Vietnam*
[e] *Academy of Finance, No. 58 Le Van Hien Street, Duc Thang Ward, Tu Liem District, Hanoi, Vietnam*

Corresponding author: Luu Quoc Dat, Datluuquoc@gmail.com

Abstract

Supplier selection and segmentation are crucial tasks of companies in order to reduce costs and increase the competitiveness of their goods. To handle uncertainty and dynamicity in the supplier segmentation problem, this research thus proposes a new dynamic generalized fuzzy multi-criteria group decision making (MCGDM) approach from the aspects of capability and willingness and with respect to environmental issues. The proposed approach defines the aggregated ratings of alternatives, the aggregated weights of criteria, and the weighted ratings by using generalized fuzzy numbers with the effect of time weight. Next, we determine the ranking order of alternatives via a popular centroid-index ranking approach. Finally, two case studies demonstrate the efficiency of the proposed dynamic approach

Keywords: Supplier segmentation, dynamic fuzzy MCGDM, centroid-index, generalized fuzzy numbers

Introduction

Supplier segmentation is a step that follows supplier selection and plays an important role in organizations for reducing production costs and optimally utilizing resources. Enterprises classify their suppliers from a selected set into distinct groups with

different needs, characteristics, and requirements in order to adopt an appropriate strategic approach for handling different supplier segments [1]. Supplier segmentation is a highly complex decision-making problem that must consider many potential criteria and decision makers under a vague environment [2,3]. Consequently, supplier segmentation can be viewed as a fuzzy multi-criteria group decision making (MCGDM) problem.

Numerous studies in the literature have proposed fuzzy multi-criteria decision making (MCDM) approaches to select and evaluate (green/sustainable) suppliers, with some recent applications found in [4-10]. While several studies used multi-criteria methods and fuzzy logic systems for solving supplier segmentation problem [2,3,11-13], existing studies on segmenting suppliers have paid limited attention to environmentally and socially related criteria [11]. Additionally, few studies have applied generalized fuzzy numbers (GFNs) to select or segment suppliers. Furthermore, they all have converted GFNs into normal fuzzy numbers through a normalization process and then applied fuzzy MCDM methods for normal fuzzy numbers. Nevertheless, the normalization process has a serious disadvantage - that is, the loss of information [14].

Chen [15] indicated in many practical situations that it is not possible to restrict the membership function to the normal form. Furthermore, the existing studies targeting supplier selection and segmentation only address static evaluation information for a certain period. However, in many real-life problems the decision makers are generally provided the information over different periods [16,17]. Lee et al. [16] proposed a dynamic fuzzy MCGDM method for performance evaluation, while Mehdi et al. [17] presented a new fuzzy dynamic MCGDM approach to assess a subcontractor. Overall, it seems that no study has yet to propose a dynamic MCGDM using GFNs for solving the green supplier segmentation (GSS) problem with the effect of a time weight.

This study primarily proposes a new dynamic generalized fuzzy MCGDM approach from the aspects of capability and willingness with respect to environmental issues. The proposed approach defines the aggregated ratings of alternatives, the aggregated weights of criteria, and the aggregated weighted ratings using GFNs with the effect of time weight. We then determine the ranking order of alternatives via a popular centroid-index ranking approach proposed by [18]. Finally, two case studies demonstrate the efficiency of the proposed approach.

Literature review on methods and criteria for supplier segmentation

This section presents an overview of the methods and criteria that have been used for supplier segmentation in the existing literature.

Supplier segmentation methods

Supplier segmentation models have been widely explored ever since the pioneering works of [19,20], who specified the variables required for segmenting suppliers [2,3,21-26]. Some of these models have been reviewed and discussed in the works of [20; 27-29]. Kraljic [20] presented a comprehensive portfolio approach to purchasing and supply segmentation. To classify materials or components, Kraljic [20] utilized two variables, the profit impact of a given item and the supply risk, under high and low levels that yield four segments: (1) non-critical items (supply risk: low; profit impact: low), (2) leverage items, (supply risk: low; profit impact: high), (3) bottleneck items (supply risk: high; profit impact: low), and (4) strategic items (supply risk: high; profit impact: high). Dyer et al. [30] developed strategic supplier segmentation based on the differences between outsourcing strategies. According to them, firms should maintain high levels of communication with suppliers that provide strategic inputs that contribute to the

differential advantage of the buyer’s final product. On the other hand, firms do not need to allocate significant resources to manage and work with suppliers that provide non-strategic inputs. Kaufman et al. [26] developed a strategic supplier typology that explains the differences in the composition and performance of various types of suppliers, using technology and collaboration to segment suppliers.

Svensson [27] applied three principal components, including the source of disturbance, the category of disturbance, and the type of logistics flow, in supplier segmentation. Hallikas et al. [24] described supplier and buyer dependency risks as the variables for classifying supplier relationships. Day et al. [28] presented the taxonomy of segmentation bases in which the buyer assesses the supply base from a purchasing perspective. Che [22] proposed two optimization mathematical models for the clustering and selection of suppliers. Model 1 is based on customer demands to cluster suppliers under a minimal total within cluster variation. Model 2 takes the results of Model 1 to determine the optimal supplier combination based on quantity discount and customer demands. Rezaei & Ortt [31] proposed a framework for classifying suppliers based on supplier capabilities and willingness. Using their framework, it is possible to segment suppliers using multiple criteria, but most existing methods are based on just two criteria.

Rezaei et al. [32] presented an approach for segmenting and developing suppliers using capabilities and willingness criteria. They employed the best worst method (BWM) to define the relative weight of the criteria and further applied a scatter plot to segment the suppliers, where the horizontal and vertical axes are capabilities and willingness, respectively. Segura & Maroto [21] utilized a hybrid MCDM approach based on PROMETHEE and Multi-Attribute Utility Theory and used Analytic Hierarchy Process (AHP) for eliciting the weights of the criteria. The authors further took historical and reliable indicators to classify suppliers. Bai et al. [11] presented a novel methodology

based on the rough set theory, VIKOR, and fuzzy C-means for green supplier segmentation, employing the dimensions of willingness and capabilities in their approach. Aineth & Ravindran [8] proposed a quantitative framework for sustainable procurement using the criteria of economic, environmental, and social hazards. Rezaei & Lajimi [33] combined purchasing portfolio matrix, supplier potential matrix, and BWM to segment suppliers. Appendix A compares the existing methods for supplier segmentation.

Supplier segmentation is a MCGDM problem that includes many criteria and decision makers within a vague environment. However, only a few studies in the literature applied the multi-criteria method and fuzzy logic systems to segment suppliers. Additionally, previous studies were limited to using normal fuzzy numbers and addressing the static evaluation information at a certain period to segment suppliers. Rezaei & Ortt [2] utilized the fuzzy AHP approach to segment suppliers using their capabilities and willingness criteria. Haghighi & Salahi [13] used the integrated fuzzy AHP approach and c-means algorithm to cluster suppliers. Akman [34] proposed a hybrid approach, including VIKOR, confirmatory factor analysis, and fuzzy c-means, to evaluate and segment suppliers in an automobile manufacturing company. The criteria of suppliers' capability and willingness were used to cluster suppliers. Lo & Sudjatmika [12] presented a modified fuzzy AHP approach for evaluating suppliers using bell-shaped membership functions. To our knowledge, no prior studies have developed the dynamic generalized fuzzy MCGDM approach with respect to environmental issues for solving supplier segmentation problem.

Green supplier segmentation criteria Use plain style

Identifying the GSS criteria is one of the main challenges of a business enterprise to formulate proper supplier segmentation. To conduct GSS, several economic, environmental, and social dimensions should be considered [6], yet the majority of prior research only considered the evaluation criteria from the economic aspect. To segment the

suppliers, our study's proposed approach takes into account not only economic criteria, but also environmental and social criteria. Appendix A summarizes the capabilities and willingness criteria drawing the greatest attention in recent literature.

Establishment of a new approach for solving green supplier selection and segmentation

This section develops a new generalized fuzzy dynamic MCGDM approach to solve the green supplier selection and segmentation problem. The procedure of the proposed approach is described as follows.

Identifying the green capabilities and willingness criteria

A committee of k decision makers $(D_v, v=1,\ldots,k)$ is assumed responsible for evaluating m suppliers $(A_i, i=1,\ldots,m)$ under n selection criteria $(C_j, j=1,\ldots,n)$ in time sequence $t_u, u=1,...,h,$ where the ratings of suppliers versus each criterion and the importance weight of the criteria are expressed by using GTFN. The criteria are classified into two categories: capabilities $(C_j, j=1,\ldots,l)$ and willingness $(C_j, j=l+1,\ldots,n).$

A dynamic MCGDM approach can be concisely expressed in matrix format as:

$$
\begin{array}{c c}
 & \begin{array}{cccc} C_1(t_u) & C_2(t_u) & \cdots & C_j(t_u) \end{array} \\
D_v(t_u)= \begin{array}{c} A_1(t_u) \\ A_2(t_u) \\ \vdots \\ A_i(t_u) \end{array} & \begin{bmatrix} x_{11}(t_u) & x_{12}(t_u) & \cdots & x_{1j}(t_u) \\ x_{21}(t_u) & x_{22}(t_u) & \cdots & x_{2j}(t_u) \\ \vdots & \vdots & \vdots & \vdots \\ x_{i1}(t_u) & x_{i2}(t_u) & \cdots & x_{ij}(t_u) \end{bmatrix}
\end{array}
$$

Aggregating the importance weights of the criteria

Let $w_{jv}(t_u) = \left\langle o_{jv}(t_u), p_{jv}(t_u), q_{jv}(t_u); \varpi_{jv}(t_u) \right\rangle, w_{jv}(t_u) \in R^*, j = 1,\ldots,n, v = 1,\ldots,k, \quad u = 1,\ldots,h,$ be the weight assigned by decision maker D_v to criterion C_j $(C_j, j = 1,\ldots,n)$ in time sequence t_u. The average weight, $w_j = (o_j, p_j, q_j; \varpi_j)$, of criterion C_j assessed by the committee of k decision makers can be evaluated as:

$$w_j = \frac{1}{h*k} \otimes \left\langle w_{j1}(t_1) \oplus w_{j2}(t_2) \oplus \ldots \oplus w_{jk}(t_u) \right\rangle, \tag{1}$$

where $o_j = \frac{1}{h*k}\sum_{v=1}^{k} o_{jv}(t_u), p_j = \frac{1}{h*k}\sum_{v=1}^{k} p_{jv}(t_u), q_j = \frac{1}{h*k}\sum_{v=1}^{k} q_{jv}(t_u)$ and $\varpi_j = \min\{\varpi_{j1}(t_1), \varpi_{j2}(t_2), \ldots, \varpi_{jk}(t_u)\}$.

Aggregating the ratings of green suppliers versus the criteria

Let $x_{ijv}(t_u) = \left\langle e_{ijv}(t_u), f_{ijv}(t_u), g_{ijv}(t_u); \varpi_{ijv}(t_u) \right\rangle, \quad i = 1,\ldots,m,\ j = 1,\ldots,n,\ v = 1,\ldots,k, \quad u = 1,\ldots,h,$ be the suitability rating assigned to supplier A_i, by decision maker D_v, for criterion C_j in time sequence t_u. The averaged suitability rating, $x_{ij} = (e_{ij}, f_{ij}, g_{ij}; \varpi_{ij}),$ can be evaluated as:

$$x_{ij} = \frac{1}{h*k} \otimes (x_{ij1}(t_1) \oplus x_{ij2}(t_2) \oplus \ldots \oplus x_{ijv}(t_u) \oplus \ldots \oplus x_{ijk}(t_h)), \tag{2}$$

where $e_{ij} = \frac{1}{h*k}\sum_{v=1}^{k} e_{ijv}(t_u), \quad f_{ij} = \frac{1}{h*k}\sum_{v=1}^{k} f_{ijv}(t_u), \quad g_{ij} = \frac{1}{h*k}\sum_{v=1}^{k} g_{ijv}(t_u)$, and $\varpi_{ij} = \min\{\varpi_{ij1}(t_1), \varpi_{ij2}(t_2), \ldots, \varpi_{ijk}(t_h)\}$.

Constructing the weighted fuzzy decision matrix

The weighted decision matrices $S_{i1} = (d_{i1}, h_{i1}, i_{i1}; \varpi_{i1})$ and $S_{i2} = (d_{i2}, h_{i2}, i_{i2}; \varpi_{i2})$ versus the capabilities $(C_j, j = 1,\ldots,l)$ and willingness criteria $(C_j, j = l+1,\ldots,n)$ in time t_u are respectively defined as follows:

$$S_{i1} = \frac{1}{l}\sum_{j=1}^{l}(s_{ij})_{m.l} = \frac{1}{l}\sum_{j=1}^{l} x_{ij} \otimes w_j,\ i = 1,\ldots,m;\ j = 1,\ldots,l, \tag{3}$$

$$S_{i2} = \frac{1}{n-l-1}\sum_{j=l+1}^{n}(s_{ij})_{m.(n-l)} = \frac{1}{n-l-1}\sum_{j=l+1}^{n} x_{ij} \otimes w_j,\ i = 1,\ldots,m;\ j = l+1,\ldots,n. \tag{4}$$

Defuzzification

This study applies the popular centroid-index ranking approach proposed by [18] to determine the ranking order of alternatives.

Segmenting the green suppliers

What was your justification to divide the suppliers?? support with evidence

Based on the distance values between the centroid and minimum points of the alternatives, we divide the suppliers into 2 × 2 segments, including Group 1 (low capabilities and low willingness), Group 2 (low capabilities and high willingness), Group 3 (high capabilities and low willingness), and Group 4 (high capabilities and high willingness). The cut-off points, which are the potential values of the distance, are determined by the decision makers; i.e., all decision makers give the linguistic variables for the ratings of alternatives as Fair = (0.3, 0.5, 0.7; 0.8).

Implementation of the proposed dynamic generalized fuzzy MCGDM approach

This section applies the proposed approach in the case of a medium-sized transport equipment company located in northern Vietnam. The managers of this company have become perplexed on how to effectively manage their suppliers to maximize their profit due to the increase in the number of suppliers. We apply the proposed approach to the process of this firm’s green supplier segmentation to help it segment its suppliers and test the efficacy of the proposed method. Data were collected by conducting semi-structured

interviews with the company's top managers and department heads (decision-makers). Three decision makers (D_1, D_2, and D_3) were requested to separately evaluate the importance weights of the capabilities and willingness criteria and the ratings of GSS at three different times (t_1, t_2, and t_3). We characterize the entire GSS procedure by the following steps.

Step 1: Aggregate the importance weights of the respective capabilities and willingness criteria.

Step 2: Aggregate the ratings of green suppliers versus capabilities and willingness criteria, respectively.

Step 3: Construct the weighted fuzzy decision matrices.

Step 4: Calculation of the distance of each green supplier.

Step 5: Segment the green suppliers.

Steps 1 and 2 were performed by the company's managers (i.e., the three decision-makers D_1, D_2, and D_3) without any intervention from the authors. Steps 3 to 5 were calculated using the proposed approach.

Aggregation of the importance weights of the respective green capabilities and willingness criteria

Following the review of the literature and discussions with the top managers and department heads, we select six capabilities (i.e., price/cost - C_1, delivery - C_2, quality - C_3, reputation and position in industry - C_4, financial position - C_5, hazardous waste management - C_6) and four willingness criteria (i.e., commitment to quality - W_1, commitment to continuous improvement in product and process - W_2, relationship closeness - W_3, willingness to share information, ideas, technology, and cost savings - W_4) for evaluating and segmenting suppliers. After determining the green suppliers' criteria,

the three company's managers are asked to define the level of importance of each criterion through a linguistic variable. Table 1 shows the aggregate weights of the criteria using Eq. (1).

Table 1. Aggregated weights of the criteria evaluated by the decision makers

Criterion	Decision maker									w_{ij}
	t_1			t_2			t_3			
	D_1	D_2	D_3	D_1	D_2	D_3	D_1	D_2	D_3	
C_1	VI	VI	VI	AI	VI	AI	AI	VI	AI	(0.633, 0.789, 0.944; 0.900)
C_2	VI	I	I	I	I	I	VI	VI	I	(0.433, 0.567, 0.700; 0.800)
C_3	VI	AI	VI	AI	VI	VI	VI	VI	VI	(0.567, 0.744, 0.922; 0.900)
C_4	VI	VI	AI	VI	VI	VI	I	VI	I	(0.511, 0.678, 0.844; 0.800)
C_5	AI	VI	VI	I	VI	I	I	VI	I	(0.489, 0.633, 0.778; 0.800)
C_6	I	VI	I	I	VI	VI	I	VI	VI	(0.456, 0.611, 0.767; 0.800)
W_1	I	I	I	VI	I	I	I	VI	I	(0.422, 0.544, 0.667; 0.800)
W_2	VI	I	VI	I	I	VI	VI	I	I	(0.444, 0.589, 0.733; 0.800)
W_3	I	I	I	I	VI	I	I	VI	I	(0.422, 0.544, 0.667; 0.800)
W_4	I	VI	I	I	VI	VI	VI	VI	I	(0.456, 0.611, 0.767; 0.800)

Aggregation of the ratings of green suppliers versus the capabilities and willingness criteria

The decision makers define the suitability ratings of twelve green suppliers (i.e., $A_1,...,A_{12}$) versus the capabilities and willingness criteria using the linguistic variables. Tables 3a to 3e (in Appendix C) present the aggregated suitability ratings of the suppliers versus the six capabilities criteria (i.e., $C_1,...,C_7$) and four willingness criteria (i.e., $W_1,...,W_6$) from the three decision makers obtained from Eq. (2) and Table 2 (in Appendix B).

Determination of the weighted rating

Table 4 shows the final fuzzy evaluation values of each green supplier using Eqs. (3) and (4).

Table 4. Final fuzzy evaluation values of each supplier

Supplier	Capabilities criteria	Willingness criteria
A_1	(0,214, 0,405, 0,653; 0,700)	(0,126, 0,262, 0,443; 0,700)
A_2	(0,124, 0,261, 0,444; 0,600)	(0,214, 0,387, 0,611; 0,800)
A_3	(0,303, 0,507, 0,762; 0,800)	(0,198, 0,372, 0,598; 0,800)
A_4	(0,131, 0,269, 0,453; 0,600)	(0,214, 0,391, 0,620; 0,800)
A_5	(0,228, 0,422, 0,674; 0,700)	(0,191, 0,358, 0,576; 0,700)
A_6	(0,231, 0,428, 0,685; 0,700)	(0,219, 0,391, 0,611; 0,800)
A_7	(0,298, 0,484, 0,716; 0,700)	(0,212, 0,386, 0,612; 0,800)
A_8	(0,137, 0,286, 0,487; 0,600)	(0,130, 0,266, 0,449; 0,600)
A_9	(0,231, 0,428, 0,683; 0,700)	(0,205, 0,377, 0,601; 0,800)
A_{10}	(0,258, 0,448, 0,692; 0,600)	(0,184, 0,353, 0,575; 0,700)
A_{11}	(0,239, 0,440, 0,699; 0,800)	(0,203, 0,378, 0,605; 0,800)
A_{12}	(0,131, 0,273, 0,464; 0,600)	(0,214, 0,378, 0,589; 0,600)

Calculation of the distance of each green supplier

We obtain the distance between the centroid point and the minimum point Go = (0,124, 0,600) of each green supplier as depicted in Table 5 by using the data in Table 4 and the ranking approach proposed by [18].

Table 5. Distance measurement

Supplier	Capabilities criteria		Willingness criteria	
	Centroid point A_i $(\bar{x}_A, \bar{y}_A)$	Distance $D(A_i, Go)$	Centroid point $A_i\,(\bar{x}_A, \bar{y}_A)$	Distance $D(A_i, Go)$
A_1	(0,424, 0,233)	0,314	(0,277, 0,233)	0,177
A_2	(0,276, 0,200)	0,172	(0,404, 0,267)	0,298
A_3	(0,524, 0,267)	0,414	(0,389, 0,267)	0,284
A_4	(0,284, 0,200)	0,179	(0,409, 0,267)	0,302
A_5	(0,442, 0,233)	0,331	(0,375, 0,233)	0,266
A_6	(0,448, 0,233)	0,338	(0,407, 0,267)	0,300
A_7	(0,499, 0,233)	0,387	(0,404, 0,267)	0,297
A_8	(0,303, 0,200)	0,197	(0,282, 0,200)	0,175
A_9	(0,447, 0,233)	0,337	(0,394, 0,267)	0,288
A_{10}	(0,466, 0,200)	0,351	(0,370, 0,233)	0,261
A_{11}	(0,459, 0,267)	0,352	(0,396, 0,267)	0,290
A_{12}	(0,289, 0,200)	0,184	(0,394, 0,200)	0,279

Segmentation of the suppliers

Based on the distance scores for the capabilities and willingness of each green supplier, we assign 12 green suppliers to one of four segments (Fig. 1) using Step 6 of the proposed methodology. In this step, the cut-off points of the green supplier's capabilities

and willingness are 0.2084 and 0.1814, respectively. Figure 1 and Table 6 show that one green supplier is assigned to Group 1, three green suppliers to Group 2, one green supplier to Group 3, and seven green suppliers to Group 4. Thus, the company has seven good green suppliers, but five of them lack capabilities, willingness, or both.

The results indicate that the company can use different strategies to handle various segments and may try and develop those green suppliers that are less capable and less willing to cooperate (i.e., Group 1 green suppliers) or terminate its relationship with them in favor of good alternatives [2,3]. Group 2 green suppliers are willing to cooperate, but are less competent to meet the buyer's requirements. The company should help these green suppliers improve their capabilities and performance or replace them with capable ones in the short term [35]. Group 3 green suppliers have high capabilities, but exhibit a low-level willingness to cooperate. The company should focus on improving its relationship with these green suppliers and determine various approaches on how to become attractive to them [36]. Group 4 green suppliers, which are the best green suppliers of the company, have great capabilities and a high level of willingness. The company should maintain a close long-term relationship with these green suppliers [31].

Table 6. Segments of the suppliers

**Segment**	**No. of suppliers**	**Supplier(s)**
Group 1	1	A_8
Group 2	3	A_2, A_4, A_{12}
Group 3	1	A_1
Group 4	7	A_3, A_5, A_6, A_7, A_{09}, A_{10}, A_{11}

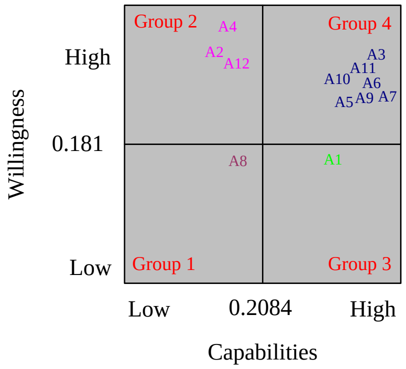

Fig. 1. Final supplier segmentation results

Comparison of the proposed method with another fuzzy MCDM method

This section compares the proposed approach in time $t_u, u = 1$ with another fuzzy MCDM approach to demonstrate its advantages and applicability by reconsidering the example investigated by [2]. In this example, a medium-sized broiler (meat-type chicken) company in the food industry intends to segment its suppliers. Six criteria for capabilities and six criteria for willingness are selected to segment 43 suppliers based on the decision makers (i.e., the managers). Table 7 shows the importance weights of the capabilities and willingness criteria.

Table 7. Importance weights of the capabilities and willingness criteria

Capabilities criterion	Fuzzy weight	Willingness criterion	Fuzzy weight
C_1^C	(0.065, 0.106, 0.181; 1.0)	C_1^W	(0.114, 0.206, 0.350; 1.0)
C_2^C	(0.110, 0.161, 0.238; 1.0)	C_2^W	(0.086, 0.150, 0.266; 1.0)
C_3^C	(0.148, 0.206, 0.279; 1.0)	C_3^W	(0.094, 0.150, 0.253; 1.0)
C_4^C	(0.115, 0.161, 0.231; 1.0)	C_4^W	(0.094, 0.150, 0.253; 1.0)
C_5^C	(0.109, 0.161, 0.240; 1.0)	C_5^W	(0.127, 0.206, 0.328; 1.0)
C_6^C	(0.132, 0.206, 0.302; 1.0)	C_6^W	(0.074, 0.137, 0.250; 1.0)

Table 8 demonstrates the averaged ratings of suppliers versus the capabilities and willingness criteria based on the data presented in Table 5 in the work of [2] and in Table 2 of this paper.

Table 8. Average ratings of suppliers versus the capabilities and willingness criteria

Supplier no.	Capabilities criteria	Willingness criteria	Supplier no.	Capabilities criteria	Willingness criteria
1	(0.037, 0.085, 0.170; 0.8)	(0.050, 0.116, 0.250; 0.8)	23	(0.051, 0.105, 0.199; 0.8)	(0.054, 0.122, 0.259; 0.8)
2	(0.051, 0.105, 0.197; 0.8)	(0.061, 0.128, 0.261; 0.8)	24	(0.024, 0.055, 0.112; 0.8)	(0.043, 0.105, 0.235; 0.8)
3	(0.052, 0.106, 0.200; 0.8)	(0.046, 0.110, 0.240; 0.8)	25	(0.039, 0.090, 0.181; 0.8)	(0.040, 0.102, 0.230; 0.8)
4	(0.058, 0.111, 0.204; 0.8)	(0.061, 0.130, 0.266; 0.8)	26	(0.037, 0.088, 0.179; 0.8)	(0.056, 0.123, 0.257; 0.8)
5	(0.041, 0.092, 0.185; 0.8)	(0.049, 0.112, 0.240; 0.8)	27	(0.046, 0.101, 0.197; 0.8)	(0.042, 0.105, 0.236; 0.8)
6	(0.039, 0.089, 0.176; 0.8)	(0.049, 0.113, 0.243; 0.8)	28	(0.058, 0.115, 0.211; 0.8)	(0.040, 0.100, 0.227; 0.8)
7	(0.056, 0.110, 0.203; 0.8)	(0.047, 0.109, 0.235; 0.8)	29	(0.033, 0.082, 0.169; 0.8)	(0.040, 0.100, 0.226; 0.8)
8	(0.063, 0.121, 0.219; 0.8)	(0.014, 0.057, 0.153; 0.8)	30	(0.019, 0.053, 0.115; 0.8)	(0.044, 0.104, 0.226; 0.8)
9	(0.017, 0.050, 0.109; 0.8)	(0.014, 0.057, 0.153; 0.8)	31	(0.039, 0.090, 0.181; 0.8)	(0.045, 0.107, 0.233; 0.8)
10	(0.017, 0.050, 0.109; 0.8)	(0.014, 0.057, 0.153; 0.8)	32	(0.052, 0.101, 0.183; 0.8)	(0.051, 0.117, 0.251; 0.8)
11	(0.043, 0.096, 0.189; 0.8)	(0.065, 0.133, 0.269; 0.8)	33	(0.045, 0.100, 0.195; 0.8)	(0.055, 0.123, 0.261; 0.8)
12	(0.048, 0.100, 0.188; 0.8)	(0.064, 0.133, 0.269; 0.8)	34	(0.046, 0.098, 0.189; 0.8)	(0.013, 0.053, 0.142; 0.8)
13	(0.054, 0.110, 0.207; 0.8)	(0.057, 0.121, 0.249; 0.8)	35	(0.046, 0.097, 0.186; 0.8)	(0.054, 0.122, 0.259; 0.8)
14	(0.031, 0.075, 0.154; 0.8)	(0.038, 0.098, 0.224; 0.8)	36	(0.039, 0.090, 0.181; 0.8)	(0.044, 0.107, 0.238; 0.8)
15	(0.043, 0.096, 0.189; 0.8)	(0.037, 0.092, 0.206; 0.8)	37	(0.061, 0.117, 0.212; 0.8)	(0.053, 0.122, 0.259; 0.8)
16	(0.025, 0.060, 0.124; 0.8)	(0.037, 0.095, 0.218; 0.8)	38	(0.044, 0.094, 0.182; 0.8)	(0.039, 0.100, 0.226; 0.8)
17	(0.025, 0.059, 0.119; 0.8)	(0.060, 0.128, 0.265; 0.8)	39	(0.038, 0.089, 0.180; 0.8)	(0.020, 0.068, 0.173; 0.8)
18	(0.014, 0.045, 0.101; 0.8)	(0.050, 0.117, 0.251; 0.8)	40	(0.047, 0.099, 0.191; 0.8)	(0.051, 0.117, 0.251; 0.8)
19	(0.052, 0.106, 0.201; 0.8)	(0.015, 0.057, 0.149; 0.8)	41	(0.032, 0.078, 0.160; 0.8)	(0.040, 0.100, 0.227; 0.8)
20	(0.039, 0.088, 0.175; 0.8)	(0.033, 0.090, 0.210; 0.8)	42	(0.053, 0.108, 0.202; 0.8)	(0.049, 0.112, 0.240; 0.8)

21	(0.019, 0.059, 0.133; 0.8)	(0.013, 0.052, 0.139; 0.8)	43	(0.031, 0.071, 0.142; 0.8)	(0.059, 0.125, 0.257; 0.8)
22	(0.048, 0.101, 0.193; 0.8)	(0.052, 0.117, 0.249; 0.8)			

We obtain the distance between the centroid and minimum points of 43 suppliers by using the ranking approach proposed by [17] as denoted in Table 9.

Table 9. Distance measurement

Supplier	Capabilities criteria			Willingness criteria		
	Centroid point	Minimum point	Distance	Centroid point	Minimum point	Distance
1	(0.097, 0.333)	(0.014, 0.333)	0,196	(0.139, 0.333)	(0.013, 0.333)	0,218
2	(0.118, 0.333)	(0.014, 0.333)	0,206	(0.150, 0.333)	(0.013, 0.333)	0,224
3	(0.119, 0.333)	(0.014, 0.333)	0,207	(0.132, 0.333)	(0.013, 0.333)	0,214
4	(0.124, 0.333)	(0.014, 0.333)	0,209	(0.153, 0.333)	(0.013, 0.333)	0,226
5	(0.106, 0.333)	(0.014, 0.333)	0,200	(0.133, 0.333)	(0.013, 0.333)	0,215
6	(0.101, 0.333)	(0.014, 0.333)	0,198	(0.135, 0.333)	(0.013, 0.333)	0,216
7	(0.123, 0.333)	(0.014, 0.333)	0,209	(0.131, 0.333)	(0.013, 0.333)	0,213
8	(0.134, 0.333)	(0.014, 0.333)	0,215	(0.074, 0.333)	(0.013, 0.333)	0,188
9	(0.059, 0.333)	(0.014, 0.333)	0,183	(0.075, 0.333)	(0.013, 0.333)	0,188
10	(0.059, 0.333)	(0.014, 0.333)	0,183	(0.075, 0.333)	(0.013, 0.333)	0,188
11	(0.109, 0.333)	(0.014, 0.333)	0,202	(0.156, 0.333)	(0.013, 0.333)	0,228
12	(0.112, 0.333)	(0.014, 0.333)	0,203	(0.155, 0.333)	(0.013, 0.333)	0,228
13	(0.124, 0.333)	(0.014, 0.333)	0,209	(0.142, 0.333)	(0.013, 0.333)	0,220
14	(0.087, 0.333)	(0.014, 0.333)	0,192	(0.120, 0.333)	(0.013, 0.333)	0,207
15	(0.109, 0.333)	(0.014, 0.333)	0,202	(0.112, 0.333)	(0.013, 0.333)	0,203
16	(0.070, 0.333)	(0.014, 0.333)	0,186	(0.117, 0.333)	(0.013, 0.333)	0,206
17	(0.068, 0.333)	(0.014, 0.333)	0,186	(0.151, 0.333)	(0.013, 0.333)	0,225
18	(0.053, 0.333)	(0.014, 0.333)	0,182	(0.139, 0.333)	(0.013, 0.333)	0,218
19	(0.120,	(0.014,	0,207	(0.074,	(0.013,	0,188

	0.333)	0.333)		0.333)	0.333)	
20	(0.101, 0.333)	(0.014, 0.333)	0,198	(0.111, 0.333)	(0.013, 0.333)	0,203
21	(0.070, 0.333)	(0.014, 0.333)	0,186	(0.068, 0.333)	(0.013, 0.333)	0,186
22	(0.114, 0.333)	(0.014, 0.333)	0,204	(0.139, 0.333)	(0.013, 0.333)	0,218
23	(0.118, 0.333)	(0.014, 0.333)	0,206	(0.145, 0.333)	(0.013, 0.333)	0,221
24	(0.064, 0.333)	(0.014, 0.333)	0,185	(0.128, 0.333)	(0.013, 0.333)	0,212
25	(0.103, 0.333)	(0.014, 0.333)	0,199	(0.124, 0.333)	(0.013, 0.333)	0,210
26	(0.102, 0.333)	(0.014, 0.333)	0,198	(0.145, 0.333)	(0.013, 0.333)	0,222
27	(0.115, 0.333)	(0.014, 0.333)	0,204	(0.128, 0.333)	(0.013, 0.333)	0,212
28	(0.128, 0.333)	(0.014, 0.333)	0,211	(0.122, 0.333)	(0.013, 0.333)	0,209
29	(0.095, 0.333)	(0.014, 0.333)	0,195	(0.122, 0.333)	(0.013, 0.333)	0,209
30	(0.062, 0.333)	(0.014, 0.333)	0,184	(0.125, 0.333)	(0.013, 0.333)	0,210
31	(0.103, 0.333)	(0.014, 0.333)	0,199	(0.128, 0.333)	(0.013, 0.333)	0,212
32	(0.112, 0.333)	(0.014, 0.333)	0,203	(0.140, 0.333)	(0.013, 0.333)	0,218
33	(0.113, 0.333)	(0.014, 0.333)	0,204	(0.146, 0.333)	(0.013, 0.333)	0,222
34	(0.111, 0.333)	(0.014, 0.333)	0,202	(0.069, 0.333)	(0.013, 0.333)	0,186
35	(0.110, 0.333)	(0.014, 0.333)	0,202	(0.145, 0.333)	(0.013, 0.333)	0,221
36	(0.103, 0.333)	(0.014, 0.333)	0,199	(0.130, 0.333)	(0.013, 0.333)	0,213
37	(0.130, 0.333)	(0.014, 0.333)	0,212	(0.145, 0.333)	(0.013, 0.333)	0,221
38	(0.107, 0.333)	(0.014, 0.333)	0,201	(0.122, 0.333)	(0.013, 0.333)	0,208
39	(0.102, 0.333)	(0.014, 0.333)	0,198	(0.087, 0.333)	(0.013, 0.333)	0,193
40	(0.112, 0.333)	(0.014, 0.333)	0,203	(0.140, 0.333)	(0.013, 0.333)	0,218
41	(0.090, 0.333)	(0.014, 0.333)	0,193	(0.122, 0.333)	(0.013, 0.333)	0,209
42	(0.121, 0.333)	(0.014, 0.333)	0,207	(0.133, 0.333)	(0.013, 0.333)	0,215
43	(0.081, 0.333)	(0.014, 0.333)	0,190	(0.147, 0.333)	(0.013, 0.333)	0,222

Based on the distance scores for the capabilities and willingness of each supplier, we assign 43 suppliers to one of four segments using Step 7 of the proposed method. The cut-off points of the supplier's capabilities and willingness are 0.196 and 0.1996, respectively. Table 10 shows that three suppliers are assigned to Group 1, nine suppliers to Group 2, three suppliers to Group 3, and twenty-eight suppliers to Group 4.

Table 10. Segments of the 43 suppliers

Segment	No. of suppliers	Suppliers
Group 1	3	A_9, A_{10}, and A_{21}
Group 2	9	A_{14}, A_{16}, A_{17}, A_{18}, A_{24}, A_{29}, A_{30}, A_{41}, andA_{43}
Group 3	3	A_{19}, A_{34}, and A_{39}
Group 4	28	A_1, A_2, A_3, A_4, A_5, A_6, A_7, A_8, A_{11}, A_{12}, A_{13}, and A_{15}, A_{20}, A_{21}, A_{22}, A_{23}, A_{25}, A_{26}, A_{27}, A_{28}, A_{31}, A_{32}, A_{33}, A_{35}, A_{36}, A_{37}, A_{38}, and A_{40}

Table 10 shows a slight difference between the segments of the 43 suppliers using the proposed method and the approach introduced by [2,3]. The reason for the difference is that the techniques proposed by [2,3] use the crisp values to measure the ratings of the suppliers. This proceeding is unreasonable, because the supplier evaluation criteria include both quantitative and qualitative criteria. The proposed method herein employs GFNs to represent the ratings of suppliers.

Discussions and Conclusions

It should be supported by literature

Green supplier segmentation (GSS) is a critical marketing activity for companies having many suppliers. Rather than formulating individual strategies for each supplier, companies can now adopt an appropriate strategic approach for handling different supplier segments. To manage the uncertainty and dynamics of GSS, this study develops a new dynamic generalized fuzzy MCGDM using capabilities and willingness criteria. The proposed approach contributes to the body of GSS literature in four significant directions. First, it expands previous studies by using GFNs instead of fuzzy numbers. Second, it is able to solve the supplier segmentation problem at different periods instead of one period.

Third, it considers not only economic criteria, but also environmental and social criteria from the aspects of suppliers' capability and willingness. Fourth, the approach can solve the GSS problem and also be employed in other management problems under similar settings.

The proposed framework uses GFNs to express the aggregated ratings of alternatives, the aggregated importance weights of criteria, and the aggregated weighted ratings with the effect of time weight. In order to rank the alternatives, we apply the most popular centroid-index ranking approach. We test the proposed approach by segmenting the suppliers of a medium-sized transport equipment company to illustrate its applicability. The company can thus formulate different strategies to handle various segments based on the outcomes obtained using the proposed method. We identify at least four major green supplier strategies: (i) maintain close long-term relationships with suppliers that have strong capabilities and high willingness; (ii) improve and attract relationships with suppliers that have high capabilities, but a low-level willingness to cooperate; (iii) help suppliers that have low capabilities, but are very willing "to green" their products and processes; (iv) terminate relationships with suppliers that are less capable and less willing to cooperate. We further compare the proposed approach with another fuzzy MCDM approach to demonstrate its superiority. Findings show that the proposed approach is an effective tool for practitioners to solve GSS problems.

The study does have some limitations. First, the proposed approach does not consider the correlation of attributes. Therefore, it is difficult to derive the weights of the decision criteria while maintaining judgment consistency. Second, by using fuzzy sets, the proposed approach cannot handle MCGDM problems that have indeterminate and inconsistent information. Future work plans are to integrate an AHP method in MCGDM

by defining the importance weights of criteria. Neutrosophic sets and their extension will also be applied to express the vague information in MCGDM.

Acknowledgements

This research is funded by “VNU University of Economics and Business, Vietnam National University, Hanoi” and “Korea Foundation for Advanced Studies (KFAS) and the Asia Research Center, Vietnam National University, Hanoi (ARC-VNU)” under project number CA.18.2A. This research was completed during and after the stay of Dr. Luu Quoc Dat at the Vietnam Institute for Advanced Study in Mathematics (VIASM).

References

[1] Parkouhi SV, Ghadikolaei AS, Lajimi HF. Resilient supplier selection and segmentation in grey environment. J Cleaner Prod 2019;207;1123-1137. https://doi.org/10.1016/j.jclepro.2018.10.007

[2] Rezaei J, Ortt R. Multi-criteria supplier segmentation using a fuzzy preference relation based AHP. Eur J Oper Res 2013; 225;75-84. https://doi.org/10.1016/j.ejor.2012.09.037

[3] Rezaei J, Ortt R. Supplier segmentation using fuzzy logic. Ind Marketing Manage 2013, 42;507-517. https://doi.org/10.1016/j.indmarman.2013.03.003

[4] Haeri SA, Rezaei J. A grey-based green supplier selection model for uncertain environments. J Cleaner Prod 2019; 221;768-784. https://doi.org/10.1016/j.jclepro.2019.02.193

[5] Hosseini S, Morshedlou N, Ivanov D, Sarder MD, Khaled AA. Resilient supplier selection and optimal order allocation under disruption risks. Int J Prod Econ 2019;213;124-137. https://doi.org/10.1016/j.ijpe.2019.03.018

[6] Yu C, Shao Y, Wang K, Zhang L. A group decision making sustainable supplier selection approach using extended TOPSIS under interval-valued Pythagorean fuzzy environment. Expert Syst Appl 2019;121;1-17. https://doi.org/10.1016/j.eswa.2018.12.010

[7] Memari K, Dargi A, Jokar MRA, Ahmad R, Rahim RA. Sustainable supplier selection: A multi-criteria intuitionistic fuzzy TOPSIS method. J Manuf Syst 2019;50;9-24. https://doi.org/10.1016/j.jmsy.2018.11.002
[8] Aineth TR, Ravindran AR. Multiple criteria framework for the sustainability risk assessment of a supplier portfolio. J Cleaner Prod 2018;172;4478-4493. https://doi.org/10.1016/j.jclepro.2017.10.304
[9] Hamdan S, Cheaitou A. Supplier selection and order allocation with green criteria: An MCDM and multi-objective optimization approach. Comput Oper Res 2017;81;282-304. https://doi.org/10.1016/j.cor.2016.11.005
[10] Yazdani M, Chatterjee P, Zavadskas EK, Zolfani SH. Integrated QFD-MCDM framework for green supplier selection. J Cleaner Prod 2017; 142; 3728-3740. https://doi.org/10.1016/j.jclepro.2016.10.095
[11] Bai C, Rezaei J, Sarkis J. Multicriteria Green Supplier Segmentation. IEEE Trans Eng Manage 2017; 64;515-528. https://doi.org/10.1109/TEM.2017.2723639
[12] Lo SC, Sudjatmika FV. Solving multi-criteria supplier segmentation based on the modified FAHP for supply chain management: a case study. Soft Comput 2016;20;4981-4990. https://doi.org/10.1007/s00500-015-1787-1
[13] Haghighi PS, Salahi MMM. Supplier Segmentation using Fuzzy Linguistic Preference Relations and Fuzzy Clustering. Intell Syst Appl 2014;05;76-82. https://doi.org/10.5815/ijisa.2014.05.08
[14] A. Kaufmann, M. Gupta, Introduction to Fuzzy Arithmetic: Theory and Applications, 2nd ed., Van Nostrand Reinhold, New York, 1991.
[15] Chen SH. Operations on fuzzy numbers with function principal. Tamkang J Manage Sci 1985; 6;13-25. https://doi.org/10.1016/S0020-0255(97)10070-6
[16] Lee J, Cho H, Kim YS. Assessing business impacts of agility criterion and order allocation strategy in multi-criteria supplier selection. Expert Syst Appl 2015;42;1136-1148. https://doi.org/10.1016/j.eswa.2014.08.041
[17] Mehdi KG, Amiri M, Zavadskas EK, Turskis Z, Antucheviciene J. A Dynamic Fuzzy Approach Based on the EDAS Method for Multi-Criteria Subcontractor Evaluation. Information 2018;9;68. https://doi.org/10.3390/info9030068.
[18] Dat LQ, Vincent FY, Chou SY. An Improved Ranking Method for Fuzzy Numbers Based on the Centroid-Index. Int J Fuzzy Syst 2011;14;413-419.
[19] Parasuraman A. Vendor segmentation: an additional level of market segmentation. Ind Marketing Manage 1980;9;59-62. https://doi.org/10.1016/S0019-8501(96)00089-2

[20] Kraljic P. Purchasing must become supply management. Harvard Bus Rev 1983;109-117.

[21] Segura M, Maroto C. A multiple criteria supplier segmentation using outranking and value function methods. Expert Syst Appl 2017;69;87-100. https://doi.org/10.1016/j.eswa.2016.10.031

[22] Che ZH. Clustering and selecting suppliers based on simulated annealing algorithms. Comput Math Appl 2012;63;228-238. https://doi.org/10.1016/j.camwa.2011.11.014

[23] Caniëls MCJ, Gelderman CJ. Power and interdependence in buyer supplier relationships: A purchasing portfolio approach. Ind Marketing Manage 2005;36;219-229. https://doi.org/10.1016/j.indmarman.2005.08.012

[24] Hallikas J, Puumalainen K, Vesterinen T, Virolainen VM. Risk-based classification of supplier relationships. J Purchasing Supply Manage 2005;11;72-82. https://doi.org/10.1016/j.pursup.2005.10.005

[25] Svensson G. Supplier segmentation in the automotive industry: A dyadic approach of a managerial model. Int J Phys Distrib Logist Manage 2004;34;12-38. https://doi.org/10.1108/09600030410515664

[26] Kaufman A, Wood CH, Theyel G. Collaboration and technology linkages: A strategic supplier typology. Strategic Manage J 2000;21;649-663. https://doi.org/10.1002/(SICI)1097-0266(200006)21:6<649::AID-SMJ108>3.0.CO;2-U

[27] Svensson G. A conceptual framework for the analysis of vulnerability in supply chains. International J Phys Distrib Logist Manage 2000;30;731-50. https://doi.org/10.1108/09600030010351444

[28] Day M, Magnan GM, Moeller MM. Evaluating the bases of supplier segmentation: A review and taxonomy. Ind Marketing Manage 2010;39;625-639. https://doi.org/10.1016/j.indmarman.2009.06.001

[29] Rezaei J, Davoodi M. A joint pricing, lot-sizing, and supplier selection model. Int J Prod Res 2012;50;4524-4542. https://doi.org/10.1080/00207543.2011.613866

[30] Dyer JH, Cho DS, Chu W. Strategic supplier segmentation: the next ‘best practice’ in supply chan management. California Manage Rev 1998;40;57-77. https://doi.org/10.2307/41165933

[31] Rezaei J, Ortt R. A multi-variable approach to supplier segmentation. Int J Prod Res 2012;50;4593-4611. https://doi.org/10.1080/00207543.2011.615352

[32] Rezaei J, Wang J, Tavasszy L. Linking supplier development to supplier segmentation using Best Worst Method. Expert Syst Appl 2015;42;9152-9164. https://doi.org/10.1016/j.eswa.2015.07.073

[33] Rezaei J, Lajimi HF. Segmenting supplies and suppliers: bringing together the purchasing portfolio matrix and the supplier potential matrix. Int J Logist Res Appl 2019;22;419-436. https://doi.org/10.1080/13675567.2018.1535649

[34] Akman G. Evaluating suppliers to include green supplier development programs via fuzzy c-means and VIKOR methods. Comput Ind Eng 2015;86;69-82. https://doi.org/10.1016/j.cie.2014.10.013

[35] Krause DR, Handfield RB, Tyler BB. The relationships between supplier development, commitment, social capital accumulation and performance improvement. J Oper Manage 2007;25;528-545. https://doi.org/10.1016/j.jom.2006.05.007

[36] Mortensen M, Arlbjørn J. Inter-organisational supplier development: The case of customer attractiveness and strategic fit. Supply Chain Manage: Int J 2012;17;152-171. https://doi.org/10.1108/13598541211212898

[37] Masella C, Rangone A. A contingent approach to the design of vendor selection systems for different types of co-operative customer/supplier relationships. Int J Oper Prod. Manage 2000;20;70-84. https://doi.org/10.1108/01443570010287044

[38] Chen SH. Ranking fuzzy numbers with maximizing set and minimizing set. Fuzzy Sets Syst 1985;17;113-129. https://doi.org/10.1016/0165-0114(85)90050-8

[39] Hsieh CH, Chen SH. Similarity of generalized fuzzy numbers with graded mean integration representation. Proc 8th International fuzzy System Association World Congress, Taipei, Taiwan, Republic of China 1999;2;551-555.

[40] Zimmermann HJ. Fuzzy Set Theory and its Applications. Kluwer Academic Publishers: Boston; 1991.

Appendices

Appendix A

Supplier segmentation approaches

Methodology	Segmentation method	Reference(s)
Conceptual	Process	[13]
	Portfolio	[18]
	Portfolio and Involvement	[37]
Empirical	Involvement	[29]
	Portfolio and Involvement	[22-25]
	AHP and Taguchi method	[21]
	AHP, Fuzzy AHP	[2,8,12,13]
	Fuzzy logic	[3]
	Portfolio, Best Worst Method	[31,32]
	Confirmatory factor analysis, VIKOR, fuzzy C-means	[11,33]
	PROMETHEE, Multi-Attribute Utility Theory	[20]

List of capabilities criteria

Criteria	Sub-criteria	Sub-sub-criteria or explanation
Economic criteria	Price/Cost	Product price, logistics cost
	Quality of products	ISO quality system, repair and return rate
	Delivery	Lead time, safety of components
	Technology	Communication and e-commerce systems, production facilities and capacity
	Flexibility	Product volume changes, using flexible machines
	Financial capability	Financial position
	Culture	Vendor’s image
	Innovativeness	New launch of products and/or technologies
	Relationship	Relationship closeness
Environ-mental criteria	Pollution production or control	Harmful materials released, pollution reduction capability, end-of-pipe controls
	Resource consumption	Consumption of resources in terms of raw material, energy, and water
	Eco-design	Design for resource efficiency, Design of products for reuse, recycle, and recovery of material
	Environmental management system	Environmental certificates, environmental implementation and operation<br>Reverse logistics system
	Green image and product	Environmental friendly product packaging, social responsibility
	Green competencies	Clean technology
	Staff environmental training	Staff training on environmental issues

Social criteria	Safety and health	Standardized health and safety conditions
	Employment practices	Job stability, employee welfare

(Sources: [4,6,7,32])

List of willingness criteria

Willingness criteria	
Commitment to quality	Open to site evaluation
Commitment to continuous improvement in product and process	Prior experience with supplier
Commitment to greening	Impression
Relationship closeness	Willingness to share: information, ideas, technology, cost savings
Honest and frequent communication	Willingness to invest in specific technology
Communication richness	Willingness to co-design
Open to site evaluation	Willingness to participate in new product development
Attitude	Willingness to eliminate waste

(Sources: [2,3,11,12])

Appendix B. Preliminaries

Generalized fuzzy numbers

We note that $H_1 = (\tilde{s}_1, \tilde{s}_2, \tilde{s}_3, \tilde{s}_4; w_{H_1}), 0 < w_{H_1} \le 1$ is a generalized trapezoidal fuzzy number (GTrFN), where $w \in (0,1]$, $\tilde{s}_1, \tilde{s}_2, \tilde{s}_3$, and $\tilde{s}_4$ are real numbers. If $w_{H_1} = 1$, then GTrFN H_1 is called a normal TrFN and denoted as $H_1 = (\tilde{s}_1, \tilde{s}_2, \tilde{s}_3, \tilde{s}_4; 1)$. If $\tilde{s}_2 = \tilde{s}_3$ then H_1 becomes a generalized triangular fuzzy number (GTFN) and can be denoted as $H_1 = (\tilde{s}_1, \tilde{s}_2, \tilde{s}_3; w_{H_1})$. The membership function $\mu_{H_1}(x)$ of GTrFN H_1 satisfies the following conditions [38, 39]:

(a) $\mu_{H_1}(x)$ is continuous to $[0, w]$;

(b) $\mu_{H_1}(x) = 0$, for all $x \in \left(-\infty, \tilde{s}_1\right]$;

(c) $\mu_{H_1}(x)$ is strictly increasing in $[\tilde{s}_1, \tilde{s}_2]$;

(d) $\mu_{H_1}(x) = w$, for all $x \in [\tilde{s}_2, \tilde{s}_3]$;

(e) $\mu_{H_1}(x)$ is strictly decreasing in $[\tilde{s}_3, \tilde{s}_4]$;

(f) $\mu_{H_1}(x) = 0,$ for all $x \in \left(\tilde{s}_4, \infty\right]$.

Arithmetic operations on generalized fuzzy numbers

Let H_1 and H_2 be two GTrFNs; i.e., $H_1 = (x_1, x_2, x_3, x_4; w_{H_1})$ and $H_2 = (y_1, y_2, y_3, y_4; w_{H_2}),$ where $x_1, x_2, x_3, x_4, y_1, y_2, y_3$ and y_4 are real values, $0 \le w_{H_1} \le 1$, and $0 \le w_{H_2} \le 1.$ Some arithmetic operators between GTrFNs H_1 and H_2 are defined as follows [38].

(i). Addition $(+)$:

$$\begin{aligned} H_1(+)H_2 &= (x_1, x_2, x_3, x_4; w_{H_1})(+)(y_1, y_2, y_3, y_4; w_{H_2}) \\ &= (x_1 + y_1, x_2 + y_2, x_3 + y_3, x_4 + y_4; \min(w_{H_1}, w_{H_2})) \end{aligned}$$

(ii). Subtraction $(-)$:

$$\begin{aligned} H_1(-)H_2 &= (x_1, x_2, x_3, x_4; w_{H_1})(-)(y_1, y_2, y_3, y_4; w_{H_2}) \\ &= (x_1 - y_4, x_2 - y_3, x_3 - y_2, x_4 - y_1; \min(w_{H_1}, w_{H_2})) \end{aligned}$$

(iii). Multiplication (x):

$$\begin{aligned} H_1(\text{x})H_2 &= (x_1, x_2, x_3, x_4; w_{H_1})(\text{x})(y_1, y_2, y_3, y_4; w_{H_2}) \\ &= (x_1 \times y_1, x_2 \times y_2, x_3 \times y_3, x_4 \times y_4; \min(w_{H_1}, w_{H_2})) \end{aligned}$$

(iv). Division $(/)$:

$$\begin{aligned} H_1(/)H_2 &= (x_1, x_2, x_3, x_4; w_{H_1})(/)(y_1, y_2, y_3, y_4; w_{H_2}) \\ &= (x_1 / y_4, x_2 / y_3, x_3 / y_2, x_4 / y_1; \min(w_{H_1}, w_{H_2})) \end{aligned}$$

Here, $x_1, x_2, x_3, x_4, y_1, y_2, y_3$ and y_4 are non-zero positive real numbers.

Linguistic variables and fuzzy numbers

Table 2 shows the linguistic variables represented by GTFNs for the ratings of alternatives and the importance weights of the criteria [40].

Table 2. Ratings of alternatives and importance weights of the criteria

Ratings		Importance weights	
Linguistic variable	GTFNs	Linguistic variable	GTFNs
Very Poor (VP)	(0.1, 0.2, 0.3; 0.6)	Unimportant (UI)	(0.0, 0.2, 0.4; 0.6)
Poor (P)	(0.2, 0.3, 0.4; 0.7)	Ordinary Important (OI)	(0.3, 0.4, 0.5; 0.7)
Fair (F)	(0.3, 0.5, 0.7; 0.8)	Important (I)	(0.4, 0.5, 0.6; 0.8)
Good (G)	(0.5, 0.7, 0.9; 0.9)	Very Important (VI)	(0.5, 0.7, 0.9; 0.9)
Very Good (VG)	(0.8, 0.9, 1.0; 1.0)	Absolutely Important (AI)	(0.8, 0.9, 1.0; 0.9)

Appendix C

Table 3a. Average ratings of suppliers versus the capabilities criteria

**Criterion**	**Supplier**	**Decision maker**									**Aggregated ratings** r_{ij}
		t_1			t_2			t_3			
		D_1	D_2	D_3	D_1	D_2	D_3	D_1	D_2	D_3	
C_1	A_1	F	F	F	P	F	P	P	F	P	(0,256, 0,411, 0,567; 0,700)
	A_2	F	P	F	F	F	F	P	F	F	(0,278, 0,456, 0,633; 0,700)
	A_3	VG	G	G	VG	G	G	G	VG	G	(0,600, 0,767, 0,933; 0,900)
	A_4	VP	P	VP	P	P	VP	P	F	P	(0,178, 0,289, 0,400; 0,600)
	A_5	G	G	G	G	G	F	VG	G	G	(0,511, 0,700, 0,889; 0,800)
	A_6	G	F	F	G	G	G	F	F	F	(0,389, 0,589, 0,789; 0,800)
	A_7	VG	VG	VG	G	VG	VG	VG	G	VG	(0,733, 0,856, 0,978; 0,900)
	A_8	VP	VP	VP	P	VP	P	P	F	F	(0,178, 0,300, 0,422; 0,600)
	A_9	G	F	G	G	G	G	G	G	G	(0,478, 0,678, 0,878; 0,800)
	A_{10}	VG	G	G	VG	G	VG	G	VG	G	(0,633, 0,789, 0,944; 0,900)
	A_{11}	F	G	F	F	G	F	F	F	F	(0,344, 0,544, 0,744; 0,800)
	A_{12}	P	P	F	F	P	F	F	P	F	(0,256, 0,411, 0,567; 0,700)
C_2	A_1	G	F	G	G	G	G	F	F	G	(0,433, 0,633, 0,833; 0,800)
	A_2	P	VP	VP	P	F	F	P	F	F	(0,222, 0,367, 0,511; 0,600)
	A_3	G	G	VG	VG	G	VG	VG	G	G	(0,633, 0,789, 0,944; 0,900)
	A_4	F	P	F	F	F	F	P	F	F	(0,278, 0,456, 0,633;

											0,700)
	A_5	F	F	P	F	F	F	F	F	G	(0,311, 0,500, 0,689; 0,700)
	A_6	G	G	G	VG	G	G	VG	G	VG	(0,600, 0,767, 0,933; 0,900)
	A_7	VG	G	VG	G	G	VG	G	G	VG	(0,633, 0,789, 0,944; 0,900)
	A_8	P	F	P	P	F	P	F	G	G	(0,300, 0,456, 0,611; 0,700)
	A_9	F	F	F	F	F	F	P	F	F	(0,289, 0,478, 0,667; 0,700)
	A_{10}	P	VP	P	P	F	F	P	F	F	(0,233, 0,378, 0,522; 0,600)
	A_{11}	G	VG	G	G	VG	G	G	G	VG	(0,600, 0,767, 0,933; 0,900)
	A_{12}	G	G	F	P	VP	P	F	P	F	(0,289, 0,444, 0,600; 0,600)

Table 3b. Average ratings of suppliers versus the capabilities criteria

**Criterion**	**Supplier**	**Decision maker**									**Aggregated ratings** r_{ij}
		t_1			t_2			t_3			
		D_1	D_2	D_3	D_1	D_2	D_3	D_1	D_2	D_3	
C_3	A_1	F	F	G	G	F	G	F	F	G	(0,389, 0,589, 0,789; 0,800)
	A_2	P	VP	P	F	F	P	P	VP	P	(0,200, 0,322, 0,444; 0,600)
	A_3	G	VG	VG	G	G	G	VG	G	G	(0,600, 0,767, 0,933; 0,900)
	A_4	F	F	F	P	F	P	P	F	P	(0,256, 0,411, 0,567; 0,700)
	A_5	F	F	G	VG	VG	G	VG	G	G	(0,556, 0,722, 0,889; 0,800)
	A_6	G	F	G	G	G	G	G	F	G	(0,456, 0,656, 0,856; 0,800)
	A_7	F	F	P	F	VG	VG	G	F	G	(0,444, 0,611, 0,778; 0,700)
	A_8	F	F	F	F	F	G	F	G	F	(0,344, 0,544, 0,744; 0,800)
	A_9	G	F	G	G	F	F	G	VG	G	(0,467, 0,656, 0,844; 0,800)
	A_{10}	F	F	F	F	F	F	G	G	VG	(0,400, 0,589, 0,778; 0,800)
	A_{11}	G	G	F	G	G	VG	G	F	F	(0,467, 0,656, 0,844; 0,800)
	A_{12}	F	F	P	P	F	F	F	P	F	(0,267, 0,433, 0,600; 0,700)
C_4	A_1	G	F	G	G	VG	G	VG	G	G	(0,544, 0,722, 0,900; 0,800)
	A_2	F	P	F	F	P	F	F	P	F	(0,267, 0,433, 0,600; 0,700)

	A_3	G	VG	G	VG	VG	G	G	G	G	(0,600, 0,767, 0,933; 0,900)
	A_4	G	F	G	P	VP	P	F	F	P	(0,289, 0,444, 0,600; 0,600)
	A_5	F	G	G	F	F	G	G	G	G	(0,433, 0,633, 0,833; 0,800)
	A_6	F	P	F	F	G	F	F	G	F	(0,333, 0,522, 0,711; 0,700)
	A_7	VG	VG	G	G	VG	VG	VG	G	G	(0,667, 0,811, 0,956; 0,900)
	A_8	P	F	P	P	F	P	F	G	F	(0,278, 0,433, 0,589; 0,700)
	A_9	F	F	F	G	F	F	G	VG	G	(0,422, 0,611, 0,800; 0,800)
	A_{10}	G	G	VG	G	F	G	VG	G	G	(0,544, 0,722, 0,900; 0,800)
	A_{11}	G	F	F	G	F	F	G	G	VG	(0,444, 0,633, 0,822; 0,800)
	A_{12}	P	VP	P	F	F	P	F	F	P	(0,233, 0,378, 0,522; 0,600)

Table 3c. Average ratings of suppliers versus the capabilities criteria

**Criterion**	**Supplier**	**Decision maker**									**Aggregated ratings** r_{ij}
		t_1			t_2			t_3			
		D_1	D_2	D_3	D_1	D_2	D_3	D_1	D_2	D_3	
C_5	A_1	G	G	F	G	VG	G	F	G	G	(0,489, 0,678, 0,867; 0,800)
	A_2	F	P	F	P	F	P	P	VP	P	(0,222, 0,356, 0,489; 0,600)
	A_3	G	VG	G	VG	G	G	F	F	G	(0,522, 0,700, 0,878; 0,800)
	A_4	G	G	G	VP	P	P	P	P	F	(0,300, 0,444, 0,589; 0,600)
	A_5	P	F	F	F	F	F	G	F	G	(0,333, 0,522, 0,711; 0,700)
	A_6	G	G	F	F	G	F	G	G	VG	(0,467, 0,656, 0,844; 0,800)
	A_7	VG	VG	VG	G	VG	G	G	G	VG	(0,667, 0,811, 0,956; 0,900)
	A_8	VP	P	VP	F	P	F	F	F	F	(0,233, 0,389, 0,544; 0,600)
	A_9	G	F	G	VG	G	G	VG	G	G	(0,544, 0,722, 0,900; 0,800)
	A_{10}	G	F	G	G	VG	G	G	G	G	(0,511, 0,700, 0,889; 0,800)
	A_{11}	G	G	G	G	G	G	G	F	G	(0,478, 0,678, 0,878; 0,800)
	A_{12}	F	P	P	F	F	P	F	F	P	(0,256, 0,411, 0,567; 0,700)
C_6	A_1	F	F	G	G	F	G	G	G	G	(0,433, 0,633, 0,833;

											0,800)
	A_2	F	P	P	F	P	F	F	P	P	(0,244, 0,389, 0,533; 0,700)
	A_3	G	G	VG	F	G	G	VG	G	VG	(0,578, 0,744, 0,911; 0,800)
	A_4	P	F	F	P	F	P	F	P	P	(0,244, 0,389, 0,533; 0,700)
	A_5	G	F	G	F	F	G	G	VG	G	(0,467, 0,656, 0,844; 0,800)
	A_6	VG	G	G	G	F	G	G	F	G	(0,489, 0,678, 0,867; 0,800)
	A_7	P	P	P	F	P	P	G	G	F	(0,289, 0,433, 0,578; 0,700)
	A_8	F	F	F	P	F	F	F	P	F	(0,278, 0,456, 0,633; 0,700)
	A_9	G	G	VG	G	F	G	G	F	F	(0,467, 0,656, 0,844; 0,800)
	A_{10}	G	VG	G	G	VG	G	VG	VG	G	(0,633, 0,789, 0,944; 0,900)
	A_{11}	G	F	G	VG	G	G	G	G	G	(0,511, 0,700, 0,889; 0,800)
	A_{12}	VP	P	VP	F	P	F	F	F	P	(0,222, 0,367, 0,511; 0,600)

Table 3d. Average ratings of suppliers versus the willingness criteria

**Criterion**	**Supplier**	**Decision maker**									**Aggregated ratings r_{ij}**
		t_1			t_2			t_3			
		D_1	D_2	D_3	D_1	D_2	D_3	D_1	D_2	D_3	
W_1	A_1	P	F	P	F	F	P	G	F	G	(0,311, 0,478, 0,644; 0,700)
	A_2	G	VG	VG	G	G	G	G	G	VG	(0,600, 0,767, 0,933; 0,900)
	A_3	G	F	G	VG	G	G	VG	G	G	(0,544, 0,722, 0,900; 0,800)
	A_4	VG	G	G	G	VG	G	G	G	G	(0,567, 0,744, 0,922; 0,900)
	A_5	G	F	G	G	G	G	F	F	G	(0,433, 0,633, 0,833; 0,800)
	A_6	G	VG	G	G	G	G	VG	G	G	(0,567, 0,744, 0,922; 0,900)
	A_7	G	G	G	F	G	G	G	F	G	(0,456, 0,656, 0,856; 0,800)
	A_8	P	F	F	P	F	P	F	G	F	(0,289, 0,456, 0,622; 0,700)
	A_9	G	G	VG	G	F	G	F	G	G	(0,489, 0,678, 0,867; 0,800)
	A_{10}	F	P	F	G	F	G	G	F	F	(0,356, 0,544, 0,733; 0,700)
	A_{11}	F	G	F	G	G	F	G	F	G	(0,411, 0,611, 0,811;

											0,800)
	A_{12}	P	VP	P	P	P	F	G	VG	G	(0,333, 0,467, 0,600; 0,600)
W_2	A_1	F	P	F	F	P	F	F	P	F	(0,267, 0,433, 0,600; 0,700)
	A_2	G	G	VG	F	G	F	G	G	VG	(0,522, 0,700, 0,878; 0,800)
	A_3	G	F	G	G	G	G	G	G	G	(0,478, 0,678, 0,878; 0,800)
	A_4	F	G	G	G	F	G	G	F	G	(0,433, 0,633, 0,833; 0,800)
	A_5	F	G	G	G	VG	G	VG	G	G	(0,544, 0,722, 0,900; 0,800)
	A_6	F	F	G	G	G	VG	G	F	G	(0,467, 0,656, 0,844; 0,800)
	A_7	G	G	VG	G	G	G	G	G	G	(0,533, 0,722, 0,911; 0,900)
	A_8	P	VP	P	P	P	F	F	F	G	(0,256, 0,400, 0,544; 0,600)
	A_9	G	G	F	G	F	F	F	G	F	(0,389, 0,589, 0,789; 0,800)
	A_{10}	G	F	F	G	G	F	G	F	G	(0,411, 0,611, 0,811; 0,800)
	A_{11}	F	G	G	G	G	G	G	G	VG	(0,511, 0,700, 0,889; 0,800)
	A_{12}	G	G	G	G	F	G	G	F	G	(0,456, 0,656, 0,856; 0,800)

Table 3e. Average ratings of suppliers versus the willingness criteria

**Criterion**	**Supplier**	**Decision maker**									**Aggregated ratings r_{ij}**
		t_1			t_2			t_3			
		D_1	D_2	D_3	D_1	D_2	D_3	D_1	D_2	D_3	
W_3	A_1	P	P	P	F	P	F	F	P	F	(0,244, 0,389, 0,533; 0,700)
	A_2	F	F	G	F	G	F	G	G	G	(0,411, 0,611, 0,811; 0,800)
	A_3	G	G	F	F	G	F	F	G	F	(0,389, 0,589, 0,789; 0,800)
	A_4	F	G	F	G	G	G	F	G	G	(0,433, 0,633, 0,833; 0,800)
	A_5	G	F	G	G	F	G	G	G	G	(0,456, 0,656, 0,856; 0,800)
	A_6	F	G	F	G	F	F	VG	G	VG	(0,478, 0,656, 0,833; 0,800)
	A_7	G	VG	VG	G	VG	G	G	G	G	(0,600, 0,767, 0,933; 0,900)
	A_8	P	P	P	F	P	F	F	F	G	(0,278, 0,433, 0,589; 0,700)
	A_9	G	VG	G	VG	VG	G	F	G	G	(0,578, 0,744, 0,911; 0,800)

	A_{10}	F	G	G	G	G	G	G	VG	G	(0,511, 0,700, 0,889; 0,800)
	A_{11}	G	G	G	G	VG	G	G	G	G	(0,533, 0,722, 0,911; 0,900)
	A_{12}	VG	G	G	G	G	G	G	G	VG	(0,567, 0,744, 0,922; 0,900)
W_4	A_1	F	F	F	P	F	F	G	F	G	(0,333, 0,522, 0,711; 0,700)
	A_2	G	G	G	G	F	G	F	G	F	(0,433, 0,633, 0,833; 0,800)
	A_3	F	G	F	G	G	F	G	G	F	(0,411, 0,611, 0,811; 0,800)
	A_4	G	VG	G	G	G	G	G	G	G	(0,533, 0,722, 0,911; 0,900)
	A_5	F	F	F	P	F	P	G	F	G	(0,322, 0,500, 0,678; 0,700)
	A_6	F	G	G	F	F	G	VG	VG	G	(0,500, 0,678, 0,856; 0,800)
	A_7	F	F	G	G	F	F	G	F	F	(0,367, 0,567, 0,767; 0,800)
	A_8	F	F	F	F	G	F	G	F	G	(0,367, 0,567, 0,767; 0,800)
	A_9	G	G	G	F	G	F	G	F	G	(0,433, 0,633, 0,833; 0,800)
	A_{10}	F	G	F	F	G	G	G	G	F	(0,411, 0,611, 0,811; 0,800)
	A_{11}	G	F	G	F	F	F	G	G	G	(0,411, 0,611, 0,811; 0,800)
	A_{12}	G	G	G	G	VG	G	VG	G	VG	(0,600, 0,767, 0,933; 0,900)

A DYNAMIC GENERALIZED FUZZY MULTI-CRITERIA GROUP DECISION MAKING APPROACH FOR GREEN SUPPLIER SEGMENTATION

Luu Huu Van[b], Do Anh Duc[a,b], Vincent F. Yu[c], Shuo-Yan Chou[c], Ngo Van Hien[d], Ngo The Chi[e], Dinh Van Toan[b], Luu Quoc Dat[b,*]

[a] *National Economics University, 207 Giai Phong Road, Hanoi*
[b] *VNU University of Economics and Business, Vietnam National University, Hanoi 144 Xuan Thuy Road, Hanoi, Vietnam*
[c] *Department of Industrial Management, National Taiwan University of Science and Technology, 43, Section 4, Keelung Road, Taipei 10607, Taiwan*
[d] *Phenikaa University, Yen Nghia Ward, Ha Dong District, Hanoi, Vietnam*
[e] *Academy of Finance, No. 58 Le Van Hien Street, Duc Thang Ward, Tu Liem District, Hanoi, Vietnam*

Corresponding author: Luu Quoc Dat, Datluuquoc@gmail.com

Abstract

Supplier selection and segmentation is aare crucial tasks of companies in order to reduce the costs and increase the competitiveness of theirfor goods. To handle the uncertainty and dynamicity of in the supplier segmentation problem, this study research thus proposes a new dynamic generalized fuzzy multi-criteria group decision making (MCGDM) approach from the aspects of capability and willingness and, with respect to environmental issues. The proposed approach defines the aggregated ratings of alternatives, the aggregated weights of criteria, and the weighted ratings by using generalized fuzzy numbers with the effect of time weight. ThenNext, we determine the ranking order of alternatives viais determined using a popular centroid-index ranking approach. Finally, two case studies were used to demonstrate the efficiency of the proposed dynamic approach

Keywords: Supplier segmentation, dynamic fuzzy MCGDM, centroid-index, generalized fuzzy numbers

Introduction

Supplier segmentation i, which is a step that follows supplier selection and, plays an important role infor organizations forto reducinge production costs and optimally

utilizing~~e~~ resources. ~~The organizations~~Enterprises classify ~~its~~ their suppliers from a selected set into distinct groups with different needs, characteristics, and requirements in order to adopt ~~the~~ an appropriate strategic approach for handling different supplier segments [1]. Supplier segmentation is a highly complex decision-making problem that must~~, which should~~ consider many potential criteria and decision makers under a vague environment [2,3]. Consequently, supplier segmentation can be viewed as a fuzzy multi-criteria group decision making (MCGDM) problem.

Numerous studies in the literature have proposed ~~the~~ fuzzy multi-criteria decision making (MCDM) approaches to select and evaluate (green/sustainable) suppliers, with s~~.~~ ~~S~~ome recent applications ~~can be~~ found in [4-10]. ~~However,~~While ~~there are~~ several studies ~~in which~~used multi-criteria methods and fuzzy logic systems ~~are used~~ for solving supplier segmentation problem [2,3,11-13], ~~. Besides,~~ existing studies on~~for~~ segmenting suppliers have paid limited attention to environmentally and socially related criteria [11]. Additionally, few ~~of~~ studies have applied ~~the~~ generalized fuzzy numbers (GFNs) to select or segment ~~the~~ suppliers. Furthermore, they all ~~of these studies~~ have converted ~~the~~ GFNs into normal fuzzy numbers through a normalization process and then applied~~y the~~ fuzzy MCDM methods for normal fuzzy numbers. Nevertheless, the normalization process has a serious disadvantage ~~-~~, that is, the loss of information [14].

Chen [15] indicated ~~that~~ in many practical situations that it is not possible to restrict the membership function to the normal form. Furthermore, the existing studies targeting~~for~~ supplier selection and segmentation only address static evaluation information for~~at~~ a certain period. However, in many real-life problems~~,~~ the decision makers are generally provided the information ~~at the~~over different periods [16,17]. Lee et al. [16] proposed a dynamic fuzzy MCGDM method for performance evaluation, while~~.~~ Mehdi et al. [17] presented a new fuzzy dynamic MCGDM approach to assess a subcontractor. Overall, i~~I~~t

seems that no study has yet to propose aies have proposed a dynamic MCGDM using the GFNs for solving the green supplier segmentation (GSS) problem with the effect of a time weight.

This study primarily aims to proposes a new dynamic generalized fuzzy MCGDM approach from the aspects of capability and willingness, with respect to environmental issues. The proposed approach defines the aggregated ratings of alternatives, the aggregated weights of criteria, and the aggregated weighted ratings using GFNs with the effect of time weight. We then determineThen, the ranking order of alternatives is determinedvia using a popular centroid-index ranking approach proposed by [18]. Finally, two case studies were used to demonstrate the efficiency of the proposed approach.

Literature review on methods and criteria for supplier segmentation

This section presents an overview of the methods and criteria which tahat have been used for supplier segmentation in the existing literature.

Supplier segmentation methods

Supplier segmentation models have been widely explored ever since the pioneering works of [19,20], who specified the variables required for segmenting suppliers [2,3,21-26]. Some of these models have been reviewed and discussed in the works of [20; 27-29]. Kraljic [20] presented a comprehensive portfolio approach to purchasing and supply segmentation. To classify the materials or components, Kraljic [20] utilizedused two variables, the namely, profit impact of a given item and the supply risk, with under high and low levels that yield four segments: (1) non-critical items (supply risk: low; profit impact: low), (2) leverage items, (supply risk: -low; profit impact: -high), (3) bottleneck

items (supply risk: -high; profit impact: -low), and (4) strategic items (supply risk: _high; profit impact: -high). Dyer et al. [30] developed a strategic supplier segmentation based on the differences between outsourcing strategies. According to them authors, firms should maintain high levels of communication with suppliers that provide strategic inputs that contribute to the differential advantage of the buyer's final product. On the other hand, firms do not need to allocate significant resources to manage and work with suppliers that provide non-strategic inputs. Kaufman et al. [26] developed a strategic supplier typology that explains the differences in the composition and performance of various types of suppliers, using t. Technology and collaboration were used to segment suppliers.

Svensson [27] applied three principal components, including the source of disturbance, the category of disturbance, and the type of logistics flow, in supplier segmentation. Hallikas et al. [24] described supplier and buyer dependency risks as the variables for classifying supplier relationships. Day et al. [28] presented the taxonomy of segmentation bases in which the buyer assesses the supply base from a purchasing perspective. Che [22] proposed two optimization mathematical models for the clustering and selection of suppliers. Model 1 iwas based on customer demands to cluster suppliers with under a minimal total within cluster variation. Model 2 takesused the results of Model 1 to determine the optimal supplier combination based on quantity discount and customer demands. Rezaei & Ortt [31] proposed a framework for classifying the suppliers based on supplier capabilities and willingness. Using their framework, it is possible to segment suppliers using multiple criteria, while most thebut most existing methods are based on just two criteria.

Rezaei et al. [32] presented an approach for segmenting and developing suppliers using capabilities and willingness criteria. They employed the bBest worst method (BWM) was employed to define the relative weight of the criteria and further applied a. A scatter

plot~~ was further applied~~ to segment the suppliers, where the horizontal and vertical axes are capabilities and willingness, respectively. Segura & Maroto [21] ~~applied~~ utilized a hybrid MCDM approach based on PROMETHEE and Multi-Attribute Utility Theory~~,~~ and used~~ing~~ Analytic Hierarchy Process (AHP) for eliciting the weights of the criteria. The authors further ~~used~~ took~~the~~ historical and reliable indicators to classify suppliers. Bai et al. [11] presented a novel methodology based on the rough set theory, VIKOR, and fuzzy C-means for green supplier segmentation, employing the dimensions of w~~. W~~illingness and capabilities ~~dimensions were used~~ in their approach. Aineth & Ravindran [8] proposed a quantitative framework for sustainable procurement using the criteria of economic, environmental, and social hazards~~ criteria~~. Rezaei & Lajimi [33] combined purchasing portfolio matrix, supplier potential matrix, and ~~the~~ BWM to segment ~~the~~ suppliers. Appendix A compares the existing methods for supplier segmentation.

Supplier segmentation is a MCGDM problem that includes many criteria and decision makers ~~under~~ within a vague environment. However, only a few studies in the literature applied the multi-criteria method and fuzzy logic systems to segment suppliers. Additionally, previous studies were limited to ~~use~~ using~~the~~ normal fuzzy numbers and addressing the static evaluation information at a certain period to segment suppliers. Rezaei & Ortt [2] ~~applied~~ utilized the fuzzy AHP approach to segment suppliers using ~~the suppliers'~~their capabilities and~~ the~~ willingness criteria. Haghighi & Salahi [13] used the integrated fuzzy AHP approach and c-means algorithm to cluster suppliers. Akman [34] proposed a hybrid approach, including VIKOR, confirmatory factor analysis, and fuzzy c-means, to evaluate and segment suppliers in an automobile manufacturing company. The criteria of suppliers' capability and willingness were used to cluster suppliers. Lo & Sudjatmika [12] presented a modified fuzzy AHP approach for evaluating suppliers using bell-shaped membership functions. To our knowledge, no prior studies have developed the

dynamic generalized fuzzy MCGDM approach with respect to environmental issues for solving supplier segmentation problem.

Green supplier segmentation criteria

Identifying~~ication of~~ the GSS criteria is one of the main challenges of a business enterprise to formulate ~~the~~ proper supplier segmentation. ~~In~~ To conduct~~ing the~~ GSS, several economic, environmental, and social dimensions should be considered [6], yet~~.~~ ~~However,~~ the majority of prior research ~~has~~ only considered the evaluation criteria from the~~in~~ economic aspect. To segment the suppliers, o~~In this~~ur study's~~, the~~ proposed approach takes into account not only economic criteria, but also environmental and social criteria ~~to segment the suppliers~~. Appendix A summarizes t~~T~~he capabilities and willingness criteria drawing the greatest attention in recent literature. ~~were summarized in the Appendix A~~

Establishment of a new approach for solving green supplier selection and segmentation

This section develops a new generalized fuzzy dynamic MCGDM approach to solve the green supplier selection and segmentation problem. The procedure of the proposed approach is described as ~~the~~ follows~~ing:~~.

Identifying the green capabilities and willingness criteria

A committee of k decision makers $(D_v, v=1,\ldots,k)$ is assumed responsible for evaluating m suppliers $(A_i, i=1,\ldots,m)$ under n selection criteria $(C_j, j=1,\ldots,n)$ in time sequence $t_u, u=1,\ldots,h,$ where the ratings of suppliers versus each criterion and the importance weight of the criteria are expressed by using GTFN. The criteria are classified

into two categories, namely,: capabilities $(C_j, j = 1,\ldots,l)$ and willingness criteria $(C_j, j = l+1,\ldots,n)$.

A dynamic MCGDM approach can be concisely expressed in matrix format as:

$$
\begin{array}{c} \\ D_v(t_u) = \end{array}
\begin{array}{c} \\ A_1(t_u) \\ A_2(t_u) \\ \vdots \\ A_i(t_u) \end{array}
\begin{array}{c} \begin{array}{cccc} C_1(t_u) & C_2(t_u) & \cdots & C_j(t_u) \end{array} \\
\begin{bmatrix} x_{11}(t_u) & x_{12}(t_u) & \cdots & x_{1j}(t_u) \\ x_{21}(t_u) & x_{22}(t_u) & \cdots & x_{2j}(t_u) \\ \vdots & \vdots & \vdots & \vdots \\ x_{i1}(t_u) & x_{i2}(t_u) & \cdots & x_{ij}(t_u) \end{bmatrix} \end{array}
$$

Aggregating the importance weights of the criteria

Let $w_{jv}(t_u) = \langle o_{jv}(t_u), p_{jv}(t_u), q_{jv}(t_u); \varpi_{jv}(t_u) \rangle, w_{jv}(t_u) \in R^*, j = 1,\ldots,n, v = 1,\ldots,k,\quad u = 1,...,h,$ be the weight assigned by decision maker D_v to criterion C_j $(C_j, j = 1,\ldots,n)$ in time sequence t_u. The average weight, $w_j = (o_j, p_j, q_j; \varpi_j)$, of criterion C_j assessed by the committee of k decision makers can be evaluated as:

$$w_j = \frac{1}{h*k} \otimes \langle w_{j1}(t_1) \oplus w_{j2}(t_2) \oplus \ldots \oplus w_{jk}(t_u) \rangle, \qquad (1)$$

Formatted: Font: (Default) Times New Roman

where $o_j = \frac{1}{h*k}\sum_{v=1}^{k} o_{jv}(t_u), p_j = \frac{1}{h*k}\sum_{v=1}^{k} p_{jv}(t_u), q_j = \frac{1}{h*k}\sum_{v=1}^{k} q_{jv}(t_u)$ and $\varpi_j = \min\{\varpi_{j1}(t_1), \varpi_{j2}(t_2), ..., \varpi_{jk}(t_u)\}$.

Aggregating the ratings of green suppliers versus the criteria

Let $x_{ijv}(t_u) = \langle e_{ijv}(t_u), f_{ijv}(t_u), g_{ijv}(t_u); \varpi_{ijv}(t_u) \rangle,\ i = 1,\ldots,m,\ j = 1,\ldots,n,\ v = 1,\ldots,k,\ u = 1,...,h,$ be the suitability rating assigned to supplier A_i, by decision maker D_v, for criterion C_j in time sequence t_u. The averaged suitability rating, $x_{ij} = (e_{ij}, f_{ij}, g_{ij}; \varpi_{ij})$, can be evaluated as:

$$x_{ij} = \frac{1}{h*k} \otimes (x_{ij1}(t_1) \oplus x_{ij2}(t_2) \oplus \ldots \oplus x_{ijv}(t_u) \oplus \ldots \oplus x_{ijk}(t_h)), \qquad (2)$$

where $e_{ij} = \frac{1}{h*k}\sum_{v=1}^{k} e_{ijv}(t_u),\quad f_{ij} = \frac{1}{h*k}\sum_{v=1}^{k} f_{ijv}(t_u),\quad g_{ij} = \frac{1}{h*k}\sum_{v=1}^{k} g_{ijv}(t_u)$, and $\varpi_{ij} = \min\{\varpi_{ij1}(t_1), \varpi_{ij2}(t_2), ..., \varpi_{ijk}(t_h)\}$.

Formatted: Font: (Default) Times New Roman

Constructing the weighted fuzzy decision matrix

The weighted decision matrices $S_{i1} = (d_{i1}, h_{i1}, i_{i1}; \varpi_{i1})$ and $S_{i2} = (d_{i2}, h_{i2}, i_{i2}; \varpi_{i2})$ versus the capabilities $(C_j, j = 1,\ldots,l)$ and willingness criteria $(C_j, j = l+1,\ldots,n)$ in time t_u are respectively defined as follows:

$$S_{i1} = \frac{1}{l}\sum_{j=1}^{l}(s_{ij})_{m.l} = \frac{1}{l}\sum_{j=1}^{l} x_{ij} \otimes w_j,\ i=1,\ldots,m;\ j=1,\ldots,l, \tag{3}$$

$$S_{i2} = \frac{1}{n-l-1}\sum_{j=l+1}^{n}(s_{ij})_{m.(n-l)} = \frac{1}{n-l-1}\sum_{j=l+1}^{n} x_{ij} \otimes w_j,\ i=1,\ldots,m;\ j=l+1,\ldots,n. \tag{4}$$

Defuzzification

This study applies the popular centroid-index ranking approach proposed by [18] to determine the ranking order of alternatives.

Segmenting the green suppliers

Based on the distance values between the centroid and minimum points of the alternatives, we divide the suppliers ~~are divided~~ into 2 × 2 segments, including Group 1 (low capabilities and low willingness), Group 2 (low capabilities and high willingness), Group 3 (high capabilities and low willingness), and Group 4 (high capabilities and high willingness). The cut-off points, which are the potential values of the distance, are determined by the decision makers;~~,~~ i.e., all decision makers give the linguistic variables for the ratings of alternatives as Fair = (0.3, 0.5, 0.7; 0.8).

Implementation of the proposed dynamic generalized fuzzy MCGDM approach

This section applies the proposed approach in the case of a medium-sized transport equipment ~~joint stock~~ company located in northern Vietnam. The managers of this company have become ~~confused~~perplexed on how to effectively manage their suppliers to maximize their profit due to ~~because of~~ the increase in the number of suppliers. We apply t~~T~~he proposed approach ~~was applied~~ to the process of this firm's green supplier segmentation ~~of this company~~ to help it segment ~~their~~ its suppliers and test the efficacy of

the proposed method. Data were collected by conducting semi-structured interviews with the company's top managers and department heads (decision-makers). Three decision makers (~~, i.e.~~ D_1, D_2, and D_3~~,~~) were requested to separately evaluate the importance weights of the capabilities and willingness criteria and the ratings of GSS at three different times (t_1, t_2, and t_3). We characterize t~~T~~he entire GSS procedure ~~was characterized~~ by the following steps.~~:~~

Step 1: Aggregate the importance weights of the respective capabilities and willingness criteria.

Step 2: Aggregate the ratings of green suppliers versus capabilities and willingness criteria, respectively.

Step 3: Construct the weighted fuzzy decision matrices.

Step 4: Calculation of the distance of each green supplier~~Defuzzify~~.

Step 5: Segment the green suppliers.

Steps 1 and 2 were performed by the company's managers (i.e., the three decision-makers~~:~~ D_1, D_2, and D_3) without any intervention from the authors. Steps 3 to 5 were calculated using the proposed approach.

Aggregation of the importance weights of the respective green capabilities and willingness criteria

Following the review of the literature and discussions with the top managers and department heads, we select six capabilities (i.e., price/cost - C_1, delivery - C_2, quality - C_3, reputation and position in industry - C_4, financial position - C_5, hazardous waste management - C_6) and four willingness criteria (i.e., commitment to quality - W_1, commitment to continuous improvement in product and process - W_2, relationship closeness - W_3, willingness to share information, ideas, technology, and cost savings - W_4)

for ~~were selected in order to~~ evaluatinge and segmenting suppliers. After determining the green suppliers' criteria, the three company's managers are asked to define the level of importance of each criterion through a linguistic variable. Table 1 shows the aggregate weights of the criteria using Eq. (1).

Table 1. Aggregated weights of the criteria evaluated by the decision makers

**Criteriona**	**Decision makers**									w_{ij}
	t_1			t_2			t_3			
	D_1	D_2	D_3	D_1	D_2	D_3	D_1	D_2	D_3	
C_1	VI	VI	VI	AI	VI	AI	AI	VI	AI	(0.633, 0.789, 0.944; 0.900)
C_2	VI	I	I	I	I	I	VI	VI	I	(0.433, 0.567, 0.700; 0.800)
C_3	VI	AI	VI	AI	VI	VI	VI	VI	VI	(0.567, 0.744, 0.922; 0.900)
C_4	VI	VI	AI	VI	VI	VI	I	VI	I	(0.511, 0.678, 0.844; 0.800)
C_5	AI	VI	VI	I	VI	I	I	VI	I	(0.489, 0.633, 0.778; 0.800)
C_6	I	VI	I	I	VI	VI	I	VI	VI	(0.456, 0.611, 0.767; 0.800)
W_1	I	I	I	VI	I	I	I	VI	I	(0.422, 0.544, 0.667; 0.800)
W_2	VI	I	VI	I	I	VI	VI	I	I	(0.444, 0.589, 0.733; 0.800)
W_3	I	I	I	I	VI	I	I	VI	I	(0.422, 0.544, 0.667; 0.800)
W_4	I	VI	I	I	VI	VI	VI	VI	I	(0.456, 0.611, 0.767; 0.800)

Aggregation of the ratings of green suppliers versus the capabilities and willingness criteria

The decision makers define the suitability ratings of twelve green suppliers (i.e., $A_1,\ldots,A_{12}$) versus the capabilities and willingness criteria using the linguistic variables. Tables 3a to 3e (in Appendix C) present the aggregated suitability ratings of the suppliers versus the six capabilities criteria (i.e., $C_1,\ldots,C_7$) and four willingness criteria (i.e., $W_1,\ldots,W_6$) from the three decision makers obtained from Eq. (2) and Table 2 (in Appendix B).

Determination of the weighted rating

Table 4 shows the final fuzzy evaluation values of each green supplier using Eqs. (3) and (4).

Table 4. Final fuzzy evaluation values of each supplier

Suppliers	Capabilities criteria	Willingness criteria
A_1	(0,214, 0,405, 0,653; 0,700)	(0,126, 0,262, 0,443; 0,700)
A_2	(0,124, 0,261, 0,444; 0,600)	(0,214, 0,387, 0,611; 0,800)
A_3	(0,303, 0,507, 0,762; 0,800)	(0,198, 0,372, 0,598; 0,800)
A_4	(0,131, 0,269, 0,453; 0,600)	(0,214, 0,391, 0,620; 0,800)
A_5	(0,228, 0,422, 0,674; 0,700)	(0,191, 0,358, 0,576; 0,700)
A_6	(0,231, 0,428, 0,685; 0,700)	(0,219, 0,391, 0,611; 0,800)
A_7	(0,298, 0,484, 0,716; 0,700)	(0,212, 0,386, 0,612; 0,800)
A_8	(0,137, 0,286, 0,487; 0,600)	(0,130, 0,266, 0,449; 0,600)
A_9	(0,231, 0,428, 0,683; 0,700)	(0,205, 0,377, 0,601; 0,800)
A_{10}	(0,258, 0,448, 0,692; 0,600)	(0,184, 0,353, 0,575; 0,700)
A_{11}	(0,239, 0,440, 0,699; 0,800)	(0,203, 0,378, 0,605; 0,800)
A_{12}	(0,131, 0,273, 0,464; 0,600)	(0,214, 0,378, 0,589; 0,600)

Calculation of the distance of each green supplier

We obtain tThe distance between the centroid point and the minimum point Go = (0,124, 0,600) of each green supplier is obtained as depicted in Table 5 by using the data in Table 4 and the ranking approach proposed by [18].

Table 5. Distance measurement

Suppliers	Capabilities criteria		Willingness criteria	
	Centroid point A_i $(\bar{x}_A, \bar{y}_A)$	Distance *D(A$_i$, Go)*	Centroid point A_i $(\bar{x}_A, \bar{y}_A)$	Distance *D(A$_i$, Go)*
A_1	(0,424, 0,233)	0,314	(0,277, 0,233)	0,177
A_2	(0,276, 0,200)	0,172	(0,404, 0,267)	0,298
A_3	(0,524, 0,267)	0,414	(0,389, 0,267)	0,284
A_4	(0,284, 0,200)	0,179	(0,409, 0,267)	0,302
A_5	(0,442, 0,233)	0,331	(0,375, 0,233)	0,266
A_6	(0,448, 0,233)	0,338	(0,407, 0,267)	0,300
A_7	(0,499, 0,233)	0,387	(0,404, 0,267)	0,297
A_8	(0,303, 0,200)	0,197	(0,282, 0,200)	0,175
A_9	(0,447, 0,233)	0,337	(0,394, 0,267)	0,288
A_{10}	(0,466, 0,200)	0,351	(0,370, 0,233)	0,261
A_{11}	(0,459, 0,267)	0,352	(0,396, 0,267)	0,290
A_{12}	(0,289, 0,200)	0,184	(0,394, 0,200)	0,279

Formatted: Font: Italic

Segmentation of the suppliers

Based on the distance scores for the capabilities and willingness of each green supplier, we assign 12 green suppliers are assigned to one of four segments (Fig. 1) using Step 6 of the proposed methodology. In this step, the cut-off points of the green supplier's

capabilities and willingness are 0.2084 and 0.1814, respectively. Figure~~.~~ 1 and Table 6 show that one green supplier is assigned to Group 1, three green suppliers ~~are assigned~~ to Group 2, one green supplier ~~is assigned~~ to Group 3, and seven green suppliers ~~are assigned~~ to Group 4. Thus, the company has seven good green suppliers, but five of them lack ~~in~~ capabilities, willingness, or both.

The results indicate that the company can use different strategies to handle various segments and ~~. The company~~ may try and develop those ~~the~~ green suppliers that are less capable and less willing to cooperate (i.e., Group 1 green suppliers) or ~~may~~ terminate its relationship with them in favor of good alternatives [2,3]. Group 2 green suppliers are willing to cooperate, but are less competent to meet the buyer's requirements. The company should help these green suppliers improve their capabilities and performance or replace them with capable ones in the short term [35]. Group 3 green suppliers have high capabilities, but exhibit~~have~~ a low-level willingness to cooperate. The company should focus on improving its relationship with these green suppliers and determine various approaches on how to become attractive to them [36]. Group 4 green suppliers, which are the best green suppliers of the company, have great capabilities and a high level of willingness. The company should maintain a close long-term relationship with these green suppliers [31].

Table 6. Segments of the suppliers

**Segment~~s~~**	**No. of suppliers**	**Supplier(s)**
Group 1	1	A_8
Group 2	3	A_2, A_4, A_{12}
Group 3	1	A_1
Group 4	7	A_3, A_5, A_6, A_7, A_{09}, A_{10}, A_{11}

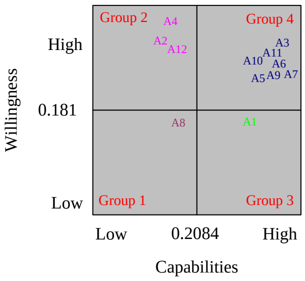

Fig. 1. Final supplier segmentation results

Comparison of the proposed method with another fuzzy MCDM method

This section compares the proposed approach in time $t_u, u=1$ with another fuzzy MCDM approach to demonstrate its advantages and applicability by reconsidering the example investigated by [2]. In this example, a medium-sized broiler (meat-type chicken) company in the food industry intends to segment ~~their~~ its suppliers. Six criteria for capabilities and six criteria for willingness are selected to segment 43 suppliers based on the decision makers (i.e., the managers). Table 7 shows the importance weights of the capabilities and willingness criteria.

Table 7. Importance weights of the capabilities and willingness criteria

**Capabilities criterion~~ia~~**	**Fuzzy weight**	**Willingness criterion~~ia~~**	**Fuzzy weight**
C_1^C	(0.065, 0.106, 0.181; 1.0)	C_1^W	(0.114, 0.206, 0.350; 1.0)
C_2^C	(0.110, 0.161, 0.238; 1.0)	C_2^W	(0.086, 0.150, 0.266; 1.0)
C_3^C	(0.148, 0.206, 0.279; 1.0)	C_3^W	(0.094, 0.150, 0.253; 1.0)
C_4^C	(0.115, 0.161, 0.231; 1.0)	C_4^W	(0.094, 0.150, 0.253; 1.0)
C_5^C	(0.109, 0.161, 0.240; 1.0)	C_5^W	(0.127, 0.206, 0.328; 1.0)
C_6^C	(0.132, 0.206, 0.302; 1.0)	C_6^W	(0.074, 0.137, 0.250; 1.0)

Table 8 demonstrates the averaged ratings of suppliers versus the capabilities and willingness criteria based on the data presented in Table 5 in the work of [2] and i~~o~~n Table 2 of~~in~~ this paper.

Table 8. Average~~d~~ ratings of suppliers versus the capabilities and willingness criteria

Supplier no.	Capabilities criteria	Willingness criteria	Supplier no.	Capabilities criteria	Willingness criteria
1	(0.037, 0.085, 0.170; 0.8)	(0.050, 0.116, 0.250; 0.8)	23	(0.051, 0.105, 0.199; 0.8)	(0.054, 0.122, 0.259; 0.8)
2	(0.051, 0.105, 0.197; 0.8)	(0.061, 0.128, 0.261; 0.8)	24	(0.024, 0.055, 0.112; 0.8)	(0.043, 0.105, 0.235; 0.8)
3	(0.052, 0.106, 0.200; 0.8)	(0.046, 0.110, 0.240; 0.8)	25	(0.039, 0.090, 0.181; 0.8)	(0.040, 0.102, 0.230; 0.8)
4	(0.058, 0.111, 0.204; 0.8)	(0.061, 0.130, 0.266; 0.8)	26	(0.037, 0.088, 0.179; 0.8)	(0.056, 0.123, 0.257; 0.8)
5	(0.041, 0.092, 0.185; 0.8)	(0.049, 0.112, 0.240; 0.8)	27	(0.046, 0.101, 0.197; 0.8)	(0.042, 0.105, 0.236; 0.8)
6	(0.039, 0.089, 0.176; 0.8)	(0.049, 0.113, 0.243; 0.8)	28	(0.058, 0.115, 0.211; 0.8)	(0.040, 0.100, 0.227; 0.8)
7	(0.056, 0.110, 0.203; 0.8)	(0.047, 0.109, 0.235; 0.8)	29	(0.033, 0.082, 0.169; 0.8)	(0.040, 0.100, 0.226; 0.8)
8	(0.063, 0.121, 0.219; 0.8)	(0.014, 0.057, 0.153; 0.8)	30	(0.019, 0.053, 0.115; 0.8)	(0.044, 0.104, 0.226; 0.8)
9	(0.017, 0.050, 0.109; 0.8)	(0.014, 0.057, 0.153; 0.8)	31	(0.039, 0.090, 0.181; 0.8)	(0.045, 0.107, 0.233; 0.8)
10	(0.017, 0.050, 0.109; 0.8)	(0.014, 0.057, 0.153; 0.8)	32	(0.052, 0.101, 0.183; 0.8)	(0.051, 0.117, 0.251; 0.8)
11	(0.043, 0.096, 0.189; 0.8)	(0.065, 0.133, 0.269; 0.8)	33	(0.045, 0.100, 0.195; 0.8)	(0.055, 0.123, 0.261; 0.8)
12	(0.048, 0.100, 0.188; 0.8)	(0.064, 0.133, 0.269; 0.8)	34	(0.046, 0.098, 0.189; 0.8)	(0.013, 0.053, 0.142; 0.8)
13	(0.054, 0.110, 0.207; 0.8)	(0.057, 0.121, 0.249; 0.8)	35	(0.046, 0.097, 0.186; 0.8)	(0.054, 0.122, 0.259; 0.8)
14	(0.031, 0.075, 0.154; 0.8)	(0.038, 0.098, 0.224; 0.8)	36	(0.039, 0.090, 0.181; 0.8)	(0.044, 0.107, 0.238; 0.8)
15	(0.043, 0.096, 0.189; 0.8)	(0.037, 0.092, 0.206; 0.8)	37	(0.061, 0.117, 0.212; 0.8)	(0.053, 0.122, 0.259; 0.8)
16	(0.025, 0.060, 0.124; 0.8)	(0.037, 0.095, 0.218; 0.8)	38	(0.044, 0.094, 0.182; 0.8)	(0.039, 0.100, 0.226; 0.8)
17	(0.025, 0.059, 0.119; 0.8)	(0.060, 0.128, 0.265; 0.8)	39	(0.038, 0.089, 0.180; 0.8)	(0.020, 0.068, 0.173; 0.8)
18	(0.014, 0.045, 0.101; 0.8)	(0.050, 0.117, 0.251; 0.8)	40	(0.047, 0.099, 0.191; 0.8)	(0.051, 0.117, 0.251; 0.8)
19	(0.052, 0.106, 0.201; 0.8)	(0.015, 0.057, 0.149; 0.8)	41	(0.032, 0.078, 0.160; 0.8)	(0.040, 0.100, 0.227; 0.8)
20	(0.039, 0.088, 0.175; 0.8)	(0.033, 0.090, 0.210; 0.8)	42	(0.053, 0.108, 0.202; 0.8)	(0.049, 0.112, 0.240; 0.8)

21	(0.019, 0.059, 0.133; 0.8)	(0.013, 0.052, 0.139; 0.8)	43	(0.031, 0.071, 0.142; 0.8)	(0.059, 0.125, 0.257; 0.8)
22	(0.048, 0.101, 0.193; 0.8)	(0.052, 0.117, 0.249; 0.8)			

We obtain t~~T~~he distance between the centroid and minimum points of 43 suppliers by ~~is obtained~~ using the ranking approach proposed by [17] as denoted in Table 9.

Table 9. Distance measurement

Supplier~~s~~	**Capabilities criteria**			**Willingness criteria**		
	Centroid point	Minimum point	Distance	Centroid point	Minimum point	Distance
1	(0.097, 0.333)	(0.014, 0.333)	0,196	(0.139, 0.333)	(0.013, 0.333)	0,218
2	(0.118, 0.333)	(0.014, 0.333)	0,206	(0.150, 0.333)	(0.013, 0.333)	0,224
3	(0.119, 0.333)	(0.014, 0.333)	0,207	(0.132, 0.333)	(0.013, 0.333)	0,214
4	(0.124, 0.333)	(0.014, 0.333)	0,209	(0.153, 0.333)	(0.013, 0.333)	0,226
5	(0.106, 0.333)	(0.014, 0.333)	0,200	(0.133, 0.333)	(0.013, 0.333)	0,215
6	(0.101, 0.333)	(0.014, 0.333)	0,198	(0.135, 0.333)	(0.013, 0.333)	0,216
7	(0.123, 0.333)	(0.014, 0.333)	0,209	(0.131, 0.333)	(0.013, 0.333)	0,213
8	(0.134, 0.333)	(0.014, 0.333)	0,215	(0.074, 0.333)	(0.013, 0.333)	0,188
9	(0.059, 0.333)	(0.014, 0.333)	0,183	(0.075, 0.333)	(0.013, 0.333)	0,188
10	(0.059, 0.333)	(0.014, 0.333)	0,183	(0.075, 0.333)	(0.013, 0.333)	0,188
11	(0.109, 0.333)	(0.014, 0.333)	0,202	(0.156, 0.333)	(0.013, 0.333)	0,228
12	(0.112, 0.333)	(0.014, 0.333)	0,203	(0.155, 0.333)	(0.013, 0.333)	0,228
13	(0.124, 0.333)	(0.014, 0.333)	0,209	(0.142, 0.333)	(0.013, 0.333)	0,220
14	(0.087, 0.333)	(0.014, 0.333)	0,192	(0.120, 0.333)	(0.013, 0.333)	0,207
15	(0.109, 0.333)	(0.014, 0.333)	0,202	(0.112, 0.333)	(0.013, 0.333)	0,203
16	(0.070, 0.333)	(0.014, 0.333)	0,186	(0.117, 0.333)	(0.013, 0.333)	0,206
17	(0.068, 0.333)	(0.014, 0.333)	0,186	(0.151, 0.333)	(0.013, 0.333)	0,225
18	(0.053, 0.333)	(0.014, 0.333)	0,182	(0.139, 0.333)	(0.013, 0.333)	0,218
19	(0.120,	(0.014,	0,207	(0.074,	(0.013,	0,188

	0.333)	0.333)		0.333)	0.333)	
20	(0.101, 0.333)	(0.014, 0.333)	0,198	(0.111, 0.333)	(0.013, 0.333)	0,203
21	(0.070, 0.333)	(0.014, 0.333)	0,186	(0.068, 0.333)	(0.013, 0.333)	0,186
22	(0.114, 0.333)	(0.014, 0.333)	0,204	(0.139, 0.333)	(0.013, 0.333)	0,218
23	(0.118, 0.333)	(0.014, 0.333)	0,206	(0.145, 0.333)	(0.013, 0.333)	0,221
24	(0.064, 0.333)	(0.014, 0.333)	0,185	(0.128, 0.333)	(0.013, 0.333)	0,212
25	(0.103, 0.333)	(0.014, 0.333)	0,199	(0.124, 0.333)	(0.013, 0.333)	0,210
26	(0.102, 0.333)	(0.014, 0.333)	0,198	(0.145, 0.333)	(0.013, 0.333)	0,222
27	(0.115, 0.333)	(0.014, 0.333)	0,204	(0.128, 0.333)	(0.013, 0.333)	0,212
28	(0.128, 0.333)	(0.014, 0.333)	0,211	(0.122, 0.333)	(0.013, 0.333)	0,209
29	(0.095, 0.333)	(0.014, 0.333)	0,195	(0.122, 0.333)	(0.013, 0.333)	0,209
30	(0.062, 0.333)	(0.014, 0.333)	0,184	(0.125, 0.333)	(0.013, 0.333)	0,210
31	(0.103, 0.333)	(0.014, 0.333)	0,199	(0.128, 0.333)	(0.013, 0.333)	0,212
32	(0.112, 0.333)	(0.014, 0.333)	0,203	(0.140, 0.333)	(0.013, 0.333)	0,218
33	(0.113, 0.333)	(0.014, 0.333)	0,204	(0.146, 0.333)	(0.013, 0.333)	0,222
34	(0.111, 0.333)	(0.014, 0.333)	0,202	(0.069, 0.333)	(0.013, 0.333)	0,186
35	(0.110, 0.333)	(0.014, 0.333)	0,202	(0.145, 0.333)	(0.013, 0.333)	0,221
36	(0.103, 0.333)	(0.014, 0.333)	0,199	(0.130, 0.333)	(0.013, 0.333)	0,213
37	(0.130, 0.333)	(0.014, 0.333)	0,212	(0.145, 0.333)	(0.013, 0.333)	0,221
38	(0.107, 0.333)	(0.014, 0.333)	0,201	(0.122, 0.333)	(0.013, 0.333)	0,208
39	(0.102, 0.333)	(0.014, 0.333)	0,198	(0.087, 0.333)	(0.013, 0.333)	0,193
40	(0.112, 0.333)	(0.014, 0.333)	0,203	(0.140, 0.333)	(0.013, 0.333)	0,218
41	(0.090, 0.333)	(0.014, 0.333)	0,193	(0.122, 0.333)	(0.013, 0.333)	0,209
42	(0.121, 0.333)	(0.014, 0.333)	0,207	(0.133, 0.333)	(0.013, 0.333)	0,215
43	(0.081, 0.333)	(0.014, 0.333)	0,190	(0.147, 0.333)	(0.013, 0.333)	0,222

Based on the distance scores for the capabilities and willingness of each supplier, we assign 43 suppliers ~~are assigned~~ to one of four segments using Step 7 of the proposed method. The cut-off points of the supplier's capabilities and willingness are 0.196 and 0.1996, respectively. Table 10 shows that three suppliers are assigned to Group 1, nine suppliers ~~are assigned~~ to Group 2, three suppliers ~~are assigned~~ to Group 3, and twenty-eight suppliers ~~are assigned~~ to Group 4.

Table 10. Segments of the 43 suppliers

Segment~~s~~	No. of suppliers	Suppliers
Group 1	3	A_9, A_{10}, and A_{21}
Group 2	9	A_{14}, A_{16}, A_{17}, A_{18}, A_{24}, A_{29}, A_{30}, A_{41}, andA_{43}
Group 3	3	A_{19}, A_{34}, and A_{39}
Group 4	28	A_1, A_2, A_3, A_4, A_5, A_6, A_7, A_8, A_{11}, A_{12}, A_{13}, and A_{15}, A_{20}, A_{21}, A_{22}, A_{23}, A_{25}, A_{26}, A_{27}, A_{28}, A_{31}, A_{32}, A_{33}, A_{35}, A_{36}, A_{37}, A_{38}, and A_{40}

Table 10 shows a slight difference between the segments of the 43 suppliers using the proposed method and the approach introduced by [2,3]. The reason for the difference is that the techniques ~~which~~ proposed by [2,3] use~~ed~~ the crisp values to measure the ratings of the suppliers. This proceeding is unreasonable, because the supplier evaluation criteria include both quantitative and qualitative criteria. T~~In t~~he proposed method herein employs~~,~~ ~~the~~ GFNs ~~were used~~ to represent the ratings of suppliers.

Discussions and Conclusions

Green supplier segmentation (~~The~~ GSS) is a critical marketing activity for~~to~~ companies ~~with~~ having many suppliers. Rather than ~~having to~~ formulating~~e~~ individual strategies for each supplier, companies can now adopt ~~the~~ an appropriate strategic approach for handling different supplier segments. To ~~handle~~manage the uncertainty and dynamics~~ity~~ of ~~the~~ GSS, this study develops a~~ed the~~ new dynamic generalized fuzzy MCGDM using capabilities and willingness criteria. The proposed approach contributes to the body of GSS literature in four significant directions. First, it expands ~~the~~ previous

studies by using GFNs instead of fuzzy numbers. Second, it is able to ~~can~~ solve the supplier segmentation problem at ~~the~~ different periods instead of one period. Third, it considers not only economic criteria, but also environmental and social criteria from the aspects of suppliers' capability and willingness. Fourth, the approach can~~not only can apply to~~ solve the GSS problem and ~~, it~~ also be~~can~~ employed in other management problems under similar settings.

~~In t~~The proposed framework uses~~approach, the~~ GFNs ~~were used~~ to express the aggregated ratings of alternatives, the aggregated importance weights of criteria, and the aggregated weighted ratings with the effect of time weight. In order to rank the alternatives, we apply the most popular centroid-index ranking approach ~~was applied~~. We test t~~T~~he proposed approach ~~was applied to~~by segmenting the suppliers of a medium-sized transport equipment ~~joint stock~~ company to illustrate its applicability. The company can thus formulate different strategies to handle various segments based on the outcomes obtained using the proposed method. We identify a~~A~~t least four major green supplier strategies ~~identified include~~: (i) maintain~~ing~~ ~~a~~ close long-term relationships with suppliers ~~who~~ that have strong capabilities and high willingness; (ii) improve~~ing~~ and attract~~ing the~~ relationships with suppliers ~~who~~ that have high capabilities, but ~~have~~ a low-level willingness to cooperate; (iii) help~~ing~~ suppliers ~~who~~ that have low capabilities, but are very willing "to green" their products and processes; (iv) terminate~~ing the~~ relationships with suppliers ~~who~~ that are less capable and less willing to cooperate. We further compare t~~T~~he proposed approach ~~was further compared~~ with another fuzzy MCDM approach to demonstrate its superiority. Findings show~~It has been demonstrated~~ that the proposed approach is an effective tool for practitioners to solve GSS problems.

The study does have~~has~~ some limitations. First~~ly~~, the proposed approach does not consider the correlation of attributes. Therefore, it is difficult to derive the weights of the

decision criteria while ~~keeping the~~maintaining judgment consistency. Second~~ly~~, by using fuzzy sets, the proposed approach cannot handle ~~the~~ MCGDM problems ~~which~~ that have ~~the~~ indeterminate~~,~~ and inconsistent information. Future work plans are to integrate an AHP method in MCGDM by defining the importance weights of criteria. Neutrosophic sets and their extension will also be applied to express the vague information in MCGDM.

Acknowledgements

This research is funded by "VNU University of Economics and Business, Vietnam National University, Hanoi" and "Korea Foundation for Advanced Studies (KFAS) and the Asia Research Center, Vietnam National University, Hanoi (ARC-VNU)" under project number CA.18.2A. This research was completed during and after the stay of ~~the seventh author~~Dr. Luu Quoc Dat at the Vietnam Institute for Advanced Study in Mathematics (VIASM).

References

[1] Parkouhi SV, Ghadikolaei AS, Lajimi HF. Resilient supplier selection and segmentation in grey environment. J Cleaner Prod 2019;207;1123-1137. https://doi.org/10.1016/j.jclepro.2018.10.007

[2] Rezaei J, Ortt R. Multi-criteria supplier segmentation using a fuzzy preference relation based AHP. Eur J Oper Res 2013; 225;75-84. https://doi.org/10.1016/j.ejor.2012.09.037

[3] Rezaei J, Ortt R. Supplier segmentation using fuzzy logic. Ind Marketing Manage 2013, 42;507-517. https://doi.org/10.1016/j.indmarman.2013.03.003

[4] Haeri SA, Rezaei J. A grey-based green supplier selection model for uncertain environments. J Cleaner Prod 2019; 221;768-784. https://doi.org/10.1016/j.jclepro.2019.02.193

[5] Hosseini S, Morshedlou N, Ivanov D, Sarder MD, Khaled AA. Resilient supplier selection and optimal order allocation under disruption risks. Int J Prod Econ 2019;213;124-137. https://doi.org/10.1016/j.ijpe.2019.03.018

[6] Yu C, Shao Y, Wang K, Zhang L. A group decision making sustainable supplier selection approach using extended TOPSIS under interval-valued Pythagorean fuzzy environment. Expert Syst Appl 2019;121;1-17. https://doi.org/10.1016/j.eswa.2018.12.010

[7] Memari K, Dargi A, Jokar MRA, Ahmad R, Rahim RA. Sustainable supplier selection: A multi-criteria intuitionistic fuzzy TOPSIS method. J Manuf Syst 2019;50;9-24. https://doi.org/10.1016/j.jmsy.2018.11.002

[8] Aineth TR, Ravindran AR. Multiple criteria framework for the sustainability risk assessment of a supplier portfolio. J Cleaner Prod 2018;172;4478-4493. https://doi.org/10.1016/j.jclepro.2017.10.304

[9] Hamdan S, Cheaitou A. Supplier selection and order allocation with green criteria: An MCDM and multi-objective optimization approach. Comput Oper Res 2017;81;282-304. https://doi.org/10.1016/j.cor.2016.11.005

[10] Yazdani M, Chatterjee P, Zavadskas EK, Zolfani SH. Integrated QFD-MCDM framework for green supplier selection. J Cleaner Prod 2017; 142; 3728-3740. https://doi.org/10.1016/j.jclepro.2016.10.095

[11] Bai C, Rezaei J, Sarkis J. Multicriteria Green Supplier Segmentation. IEEE Trans Eng Manage 2017; 64;515-528. https://doi.org/10.1109/TEM.2017.2723639

[12] Lo SC, Sudjatmika FV. Solving multi-criteria supplier segmentation based on the modified FAHP for supply chain management: a case study. Soft Comput 2016;20;4981-4990. https://doi.org/10.1007/s00500-015-1787-1

[13] Haghighi PS, Salahi MMM. Supplier Segmentation using Fuzzy Linguistic Preference Relations and Fuzzy Clustering. Intell Syst Appl 2014;05;76-82. https://doi.org/10.5815/ijisa.2014.05.08

[14] A. Kaufmann, M. Gupta, Introduction to Fuzzy Arithmetic: Theory and Applications, 2nd ed., Van Nostrand Reinhold, New York, 1991.

[15] Chen SH. Operations on fuzzy numbers with function principal. Tamkang J Manage Sci 1985; 6;13-25. https://doi.org/10.1016/S0020-0255(97)10070-6

[16] Lee J, Cho H, Kim YS. Assessing business impacts of agility criterion and order allocation strategy in multi-criteria supplier selection. Expert Syst Appl 2015;42;1136-1148. https://doi.org/10.1016/j.eswa.2014.08.041

[17] Mehdi KG, Amiri M, Zavadskas EK, Turskis Z, Antucheviciene J. A Dynamic Fuzzy Approach Based on the EDAS Method for Multi-Criteria Subcontractor Evaluation. Information 2018;9;68. https://doi.org/10.3390/info9030068.

[18] Dat LQ, Vincent FY, Chou SY. An Improved Ranking Method for Fuzzy Numbers Based on the Centroid-Index. Int J Fuzzy Syst 2011;14;413-419.

[19] Parasuraman A. Vendor segmentation: an additional level of market segmentation. Ind Marketing Manage 1980;9;59-62. https://doi.org/10.1016/S0019-8501(96)00089-2

[20] Kraljic P. Purchasing must become supply management. Harvard Bus Rev 1983;109-117.

[21] Segura M, Maroto C. A multiple criteria supplier segmentation using outranking and value function methods. Expert Syst Appl 2017;69;87-100. https://doi.org/10.1016/j.eswa.2016.10.031

[22] Che ZH. Clustering and selecting suppliers based on simulated annealing algorithms. Comput Math Appl 2012;63;228-238. https://doi.org/10.1016/j.camwa.2011.11.014

[23] Caniëls MCJ, Gelderman CJ. Power and interdependence in buyer supplier relationships: A purchasing portfolio approach. Ind Marketing Manage 2005;36;219-229. https://doi.org/10.1016/j.indmarman.2005.08.012

[24] Hallikas J, Puumalainen K, Vesterinen T, Virolainen VM. Risk-based classification of supplier relationships. J Purchasing Supply Manage 2005;11;72-82. https://doi.org/10.1016/j.pursup.2005.10.005

[25] Svensson G. Supplier segmentation in the automotive industry: A dyadic approach of a managerial model. Int J Phys Distrib Logist Manage 2004;34;12-38. https://doi.org/10.1108/09600030410515664

[26] Kaufman A, Wood CH, Theyel G. Collaboration and technology linkages: A strategic supplier typology. Strategic Manage J 2000;21;649-663. https://doi.org/10.1002/(SICI)1097-0266(200006)21:6<649::AID-SMJ108>3.0.CO;2-U

[27] Svensson G. A conceptual framework for the analysis of vulnerability in supply chains. International J Phys Distrib Logist Manage 2000;30;731-50. https://doi.org/10.1108/09600030010351444

[28] Day M, Magnan GM, Moeller MM. Evaluating the bases of supplier segmentation: A review and taxonomy. Ind Marketing Manage 2010;39;625-639. https://doi.org/10.1016/j.indmarman.2009.06.001

[29] Rezaei J, Davoodi M. A joint pricing, lot-sizing, and supplier selection model. Int J Prod Res 2012;50;4524-4542. https://doi.org/10.1080/00207543.2011.613866

[30] Dyer JH, Cho DS, Chu W. Strategic supplier segmentation: the next ‘best practice’ in supply chan management. California Manage Rev 1998;40;57-77. https://doi.org/10.2307/41165933

[31] Rezaei J, Ortt R. A multi-variable approach to supplier segmentation. Int J Prod Res 2012;50;4593-4611. https://doi.org/10.1080/00207543.2011.615352

[32] Rezaei J, Wang J, Tavasszy L. Linking supplier development to supplier segmentation using Best Worst Method. Expert Syst Appl 2015;42;9152-9164. https://doi.org/10.1016/j.eswa.2015.07.073

[33] Rezaei J, Lajimi HF. Segmenting supplies and suppliers: bringing together the purchasing portfolio matrix and the supplier potential matrix. Int J Logist Res Appl 2019;22;419-436. https://doi.org/10.1080/13675567.2018.1535649

[34] Akman G. Evaluating suppliers to include green supplier development programs via fuzzy c-means and VIKOR methods. Comput Ind Eng 2015;86;69-82. https://doi.org/10.1016/j.cie.2014.10.013

[35] Krause DR, Handfield RB, Tyler BB. The relationships between supplier development, commitment, social capital accumulation and performance improvement. J Oper Manage 2007;25;528-545. https://doi.org/10.1016/j.jom.2006.05.007

[36] Mortensen M, Arlbjørn J. Inter-organisational supplier development: The case of customer attractiveness and strategic fit. Supply Chain Manage: Int J 2012;17;152-171. https://doi.org/10.1108/13598541211212898

[37] Masella C, Rangone A. A contingent approach to the design of vendor selection systems for different types of co-operative customer/supplier relationships. Int J Oper Prod. Manage 2000;20;70-84. https://doi.org/10.1108/01443570010287044

[38] Chen SH. Ranking fuzzy numbers with maximizing set and minimizing set. Fuzzy Sets Syst 1985;17;113-129. https://doi.org/10.1016/0165-0114(85)90050-8

[39] Hsieh CH, Chen SH. Similarity of generalized fuzzy numbers with graded mean integration representation. Proc 8th International fuzzy System Association World Congress, Taipei, Taiwan, Republic of China 1999;2;551-555.

[40] Zimmermann HJ. Fuzzy Set Theory and its Applications. Kluwer Academic Publishers: Boston; 1991.

Appendices

Appendix A

Supplier segmentation approaches

Methodology	Segmentation method	Reference(s)
Conceptual	Process	[13]
	Portfolio	[18]
	Portfolio and Involvement	[37]
Empirical	Involvement	[29]
	Portfolio and Involvement	[22-25]
	AHP and Taguchi method	[21]
	AHP, Fuzzy AHP	[2,8,12,13]
	Fuzzy logic	[3]
	Portfolio, Best Worst Method	[31,32]
	Confirmatory factor analysis, VIKOR, fuzzy C-means	[11,33]
	PROMETHEE, Multi-Attribute Utility Theory	[20]

List of capabilities criteria

Criteria	Sub-criteria	Sub-sub-criteria or explanation
Economic criteria	Price/Cost	Product price, logistics cost
	Quality of products	ISO quality system, repair and return rate
	Delivery	Lead time, safety of components
	Technology	Communication and e-commerce systems, production facilities and capacity
	Flexibility	Product volume changes, using flexible machines
	Financial capability	Financial position
	Culture	Vendor's image
	Innovativeness	New launch of products and/or technologies
	Relationship	Relationship closeness
Environ-mental criteria	Pollution production or control	Harmful materials released, pollution reduction capability, end-of-pipe controls
	Resource consumption	Consumption of resources in terms of raw material, energy, and water
	Eco-design	Design for resource efficiency, Design of products for reuse, recycle, and recovery of material

	Environmental management system	Environmental certificates, environmental implementation and operation<br>Reverse logistics system
	Green image and product	Environmental friendly product packaging, social responsibility
	Green competencies	Clean technology
	Staff environmental training	Staff training on environmental issues
Social criteria	Safety and health	Standardized health and safety conditions
	Employment practices	Job stability, employee welfare

(Sources: [4,6,7,32])

List of willingness criteria

Willingness criteria	
Commitment to quality	Open to site evaluation
Commitment to continuous improvement in product and process	Prior experience with supplier
Commitment to greening	Impression
Relationship closeness	Willingness to share: information, ideas, technology, cost savings
Honest and frequent communication	Willingness to invest in specific technology
Communication richness	Willingness to co-design
Open to site evaluation	Willingness to participate in new product development
Attitude	Willingness to eliminate waste

(Sources: [2,3,11,12])

Appendix B. Preliminaries

Generalized fuzzy numbers

We note that $H_1 = (\tilde{s}_1, \tilde{s}_2, \tilde{s}_3, \tilde{s}_4; w_{H_1}), 0 < w_{H_1} \le 1$ is a generalized trapezoidal fuzzy number (GTrFN), where $w \in (0,1]$, $\tilde{s}_1, \tilde{s}_2, \tilde{s}_3$ -, and $\tilde{s}_4$ are real numbers. If $w_{H_1} = 1$, then the GTrFN H_1 is called a normal TrFN and denoted as $H_1 = (\tilde{s}_1, \tilde{s}_2, \tilde{s}_3, \tilde{s}_4; 1)$. If $\tilde{s}_2 = \tilde{s}_3$ then the H_1 becomes a generalized triangular fuzzy number (GTFN), and can be denoted as $H_1 = (\tilde{s}_1, \tilde{s}_2, \tilde{s}_3; w_{H_1})$. The membership function $\mu_{H_1}(x)$ of the GTrFN H_1 satisfies the following conditions [38, 39]:

Formatted: Font: (Default) Times New Roman

(a) $\mu_{H_1}(x)$ is a continuous to $[0, w]$;

(b) $\mu_{H_1}(x) = 0$ f, for all $x \in \left(-\infty, \tilde{s}_1\right]$;

(c) $\mu_{H_1}(x)$ is strictly increasing i~~o~~n $[\tilde{s}_1, \tilde{s}_2]$;

(d) $\mu_{H_1}(x) = w,$ for all $x \in [\tilde{s}_2, \tilde{s}_3]$;

(e) $\mu_{H_1}(x)$ is strictly decreasing i~~o~~n $[\tilde{s}_3, \tilde{s}_4]$;

(f) $\mu_{H_1}(x) = 0,$ for all $x \in \left(\tilde{s}_4, \infty\right]$.

Arithmetic operations on generalized fuzzy numbers

Let H_1 and H_2 be ~~are~~ two GTrFNs;~~,~~ i.e., $H_1 = (x_1, x_2, x_3, x_4; w_{H_1})$ and $H_2 = (y_1, y_2, y_3, y_4; w_{H_2})$, where $x_1, x_2, x_3, x_4, y_1, y_2, y_3$ and y_4 are real values, $0 \le w_{H_1} \le 1$ ~~,~~ and $0 \le w_{H_2} \le 1.$ Some arithmetic operators between ~~the~~ GTrFNs H_1 and H_2 are defined as follows [38].~~:~~

(i). Addition $(+)$:

$$H_1(+)H_2 = (x_1, x_2, x_3, x_4; w_{H_1})(+)(y_1, y_2, y_3, y_4; w_{H_2})$$
$$= (x_1 + y_1, x_2 + y_2, x_3 + y_3, x_4 + y_4; \min(w_{H_1}, w_{H_2}))$$

(ii). Subtraction $(-)$:

$$H_1(-)H_2 = (x_1, x_2, x_3, x_4; w_{H_1})(-)(y_1, y_2, y_3, y_4; w_{H_2})$$
$$= (x_1 - y_4, x_2 - y_3, x_3 - y_2, x_4 - y_1; \min(w_{H_1}, w_{H_2}))$$

(iii). Multiplication (x):

$$H_1(\mathrm{x})H_2 = (x_1, x_2, x_3, x_4; w_{H_1})(\mathrm{x})(y_1, y_2, y_3, y_4; w_{H_2})$$
$$= (x_1 \times y_1, x_2 \times y_2, x_3 \times y_3, x_4 \times y_4; \min(w_{H_1}, w_{H_2}))$$

(iv). Division $(/)$:

$$H_1(/)H_2 = (x_1, x_2, x_3, x_4; w_{H_1})(/)(y_1, y_2, y_3, y_4; w_{H_2})$$
$$= (x_1 / y_4, x_2 / y_3, x_3 / y_2, x_4 / y_1; \min(w_{H_1}, w_{H_2}))$$

Here, ~~where~~ $x_1, x_2, x_3, x_4, y_1, y_2, y_3$ and y_4 ~~b~~are non-zero positive real numbers.

Linguistic variables and fuzzy numbers

Formatted: Font: (Default) Times New Roman

Table 2 shows the linguistic variables representeding by GTFNs forof the ratings of alternatives and the importance weights of the criteria [40].

Table 2. Ratings of alternatives and importance weights of the criteria

**Ratings**		**Importance weights**	
Linguistic variables	GTFNs	Linguistic variables	GTFNs
Very Poor (VP)	(0.1, 0.2, 0.3; 0.6)	Unimportant (UI)	(0.0, 0.2, 0.4; 0.6)
Poor (P)	(0.2, 0.3, 0.4; 0.7)	Ordinary Important (OI)	(0.3, 0.4, 0.5; 0.7)
Fair (F)	(0.3, 0.5, 0.7; 0.8)	Important (I)	(0.4, 0.5, 0.6; 0.8)
Good (G)	(0.5, 0.7, 0.9; 0.9)	Very Important (VI)	(0.5, 0.7, 0.9; 0.9)
Very Good (VG)	(0.8, 0.9, 1.0; 1.0)	Absolutely Important (AI)	(0.8, 0.9, 1.0; 0.9)

Appendix C

Table 3a. Averaged ratings of suppliers versus the capabilities criteria

**Criterion**	**Suppliers**	**Decision makers**									**Aggregated ratings r_{ij}**
		t_1			t_2			t_3			
		D_1	D_2	D_3	D_1	D_2	D_3	D_1	D_2	D_3	
C_1	A_1	F	F	F	P	F	P	P	F	P	(0,256, 0,411, 0,567; 0,700)
	A_2	F	P	F	F	F	F	P	F	F	(0,278, 0,456, 0,633; 0,700)
	A_3	VG	G	G	VG	G	G	G	VG	G	(0,600, 0,767, 0,933; 0,900)
	A_4	VP	P	VP	P	P	VP	P	F	P	(0,178, 0,289, 0,400; 0,600)
	A_5	G	G	G	G	G	F	VG	G	G	(0,511, 0,700, 0,889; 0,800)
	A_6	G	F	F	G	G	G	F	F	F	(0,389, 0,589, 0,789; 0,800)
	A_7	VG	VG	VG	G	VG	VG	VG	G	VG	(0,733, 0,856, 0,978; 0,900)
	A_8	VP	VP	VP	P	VP	P	P	F	F	(0,178, 0,300, 0,422; 0,600)
	A_9	G	F	G	G	G	G	G	G	G	(0,478, 0,678, 0,878; 0,800)
	A_{10}	VG	G	G	VG	G	VG	G	VG	G	(0,633, 0,789, 0,944; 0,900)
	A_{11}	F	G	F	F	G	F	F	F	F	(0,344, 0,544, 0,744; 0,800)
	A_{12}	P	P	F	F	P	F	F	P	F	(0,256, 0,411, 0,567; 0,700)
C_2	A_1	G	F	G	G	G	G	F	F	G	(0,433, 0,633, 0,833; 0,800)
	A_2	P	VP	VP	P	F	F	P	F	F	(0,222, 0,367, 0,511; 0,600)
	A_3	G	G	VG	VG	G	VG	VG	G	G	(0,633, 0,789, 0,944; 0,900)
	A_4	F	P	F	F	F	F	P	F	F	(0,278, 0,456, 0,633;

Formatted Table

											0,700)
	A_5	F	F	P	F	F	F	F	F	G	(0,311, 0,500, 0,689; 0,700)
	A_6	G	G	G	VG	G	G	VG	G	VG	(0,600, 0,767, 0,933; 0,900)
	A_7	VG	G	VG	G	G	VG	G	G	VG	(0,633, 0,789, 0,944; 0,900)
	A_8	P	F	P	P	F	P	F	G	G	(0,300, 0,456, 0,611; 0,700)
	A_9	F	F	F	F	F	F	P	F	F	(0,289, 0,478, 0,667; 0,700)
	A_{10}	P	VP	P	P	F	F	P	F	F	(0,233, 0,378, 0,522; 0,600)
	A_{11}	G	VG	G	G	VG	G	G	G	VG	(0,600, 0,767, 0,933; 0,900)
	A_{12}	G	G	F	P	VP	P	F	P	F	(0,289, 0,444, 0,600; 0,600)

Table 3b. -Average~~d~~ ratings of suppliers versus the capabilities criteria

**Criterion~~a~~**	**Suppliers**	**Decision makers**									**Aggregated ratings** r_{ij}
		t_1			t_2			t_3			
		D_1	D_2	D_3	D_1	D_2	D_3	D_1	D_2	D_3	
C_3	A_1	F	F	G	G	F	G	F	F	G	(0,389, 0,589, 0,789; 0,800)
	A_2	P	VP	P	F	F	P	P	VP	P	(0,200, 0,322, 0,444; 0,600)
	A_3	G	VG	VG	G	G	G	VG	G	G	(0,600, 0,767, 0,933; 0,900)
	A_4	F	F	F	P	F	P	P	F	P	(0,256, 0,411, 0,567; 0,700)
	A_5	F	F	G	VG	VG	G	VG	G	G	(0,556, 0,722, 0,889; 0,800)
	A_6	G	F	G	G	G	G	G	F	G	(0,456, 0,656, 0,856; 0,800)
	A_7	F	F	P	F	VG	VG	G	F	G	(0,444, 0,611, 0,778; 0,700)
	A_8	F	F	F	F	F	G	F	G	F	(0,344, 0,544, 0,744; 0,800)
	A_9	G	F	G	G	F	F	G	VG	G	(0,467, 0,656, 0,844; 0,800)
	A_{10}	F	F	F	F	F	F	G	G	VG	(0,400, 0,589, 0,778; 0,800)
	A_{11}	G	G	F	G	G	VG	G	F	F	(0,467, 0,656, 0,844; 0,800)
	A_{12}	F	F	P	P	F	F	F	P	F	(0,267, 0,433, 0,600; 0,700)
C_4	A_1	G	F	G	G	VG	G	VG	G	G	(0,544, 0,722, 0,900; 0,800)
	A_2	F	P	F	F	P	F	F	P	F	(0,267, 0,433, 0,600; 0,700)

Formatted Table

	A_3	G	VG	G	VG	VG	G	G	G	G	(0,600, 0,767, 0,933; 0,900)
	A_4	G	F	G	P	VP	P	F	F	P	(0,289, 0,444, 0,600; 0,600)
	A_5	F	G	G	F	F	G	G	G	G	(0,433, 0,633, 0,833; 0,800)
	A_6	F	P	F	F	G	F	F	G	F	(0,333, 0,522, 0,711; 0,700)
	A_7	VG	VG	G	G	VG	VG	VG	G	G	(0,667, 0,811, 0,956; 0,900)
	A_8	P	F	P	P	F	P	F	G	F	(0,278, 0,433, 0,589; 0,700)
	A_9	F	F	F	G	F	F	G	VG	G	(0,422, 0,611, 0,800; 0,800)
	A_{10}	G	G	VG	G	F	G	VG	G	G	(0,544, 0,722, 0,900; 0,800)
	A_{11}	G	F	F	G	F	F	G	G	VG	(0,444, 0,633, 0,822; 0,800)
	A_{12}	P	VP	P	F	F	P	F	F	P	(0,233, 0,378, 0,522; 0,600)

Table 3c. Averaged ratings of suppliers versus the capabilities criteria

Criterion	Suppliers	Decision makers									Aggregated ratings r_{ij}
		t_1			t_2			t_3			
		D_1	D_2	D_3	D_1	D_2	D_3	D_1	D_2	D_3	
C_5	A_1	G	G	F	G	VG	G	F	G	G	(0,489, 0,678, 0,867; 0,800)
	A_2	F	P	F	P	F	P	P	VP	P	(0,222, 0,356, 0,489; 0,600)
	A_3	G	VG	G	VG	G	G	F	F	G	(0,522, 0,700, 0,878; 0,800)
	A_4	G	G	G	VP	P	P	P	P	F	(0,300, 0,444, 0,589; 0,600)
	A_5	P	F	F	F	F	F	G	F	G	(0,333, 0,522, 0,711; 0,700)
	A_6	G	G	F	F	G	F	G	G	VG	(0,467, 0,656, 0,844; 0,800)
	A_7	VG	VG	VG	G	VG	G	G	G	VG	(0,667, 0,811, 0,956; 0,900)
	A_8	VP	P	VP	F	P	F	F	F	F	(0,233, 0,389, 0,544; 0,600)
	A_9	G	F	G	VG	G	G	VG	G	G	(0,544, 0,722, 0,900; 0,800)
	A_{10}	G	F	G	G	VG	G	G	G	G	(0,511, 0,700, 0,889; 0,800)
	A_{11}	G	G	G	G	G	G	G	F	G	(0,478, 0,678, 0,878; 0,800)
	A_{12}	F	P	P	F	F	P	F	F	P	(0,256, 0,411, 0,567; 0,700)
C_6	A_1	F	F	G	G	F	G	G	G	G	(0,433, 0,633, 0,833;

											0,800)
	A_2	F	P	P	F	P	F	F	P	P	(0,244, 0,389, 0,533; 0,700)
	A_3	G	G	VG	F	G	G	VG	G	VG	(0,578, 0,744, 0,911; 0,800)
	A_4	P	F	F	P	F	P	F	P	P	(0,244, 0,389, 0,533; 0,700)
	A_5	G	F	G	F	F	G	G	VG	G	(0,467, 0,656, 0,844; 0,800)
	A_6	VG	G	G	G	F	G	G	F	G	(0,489, 0,678, 0,867; 0,800)
	A_7	P	P	P	F	P	P	G	G	F	(0,289, 0,433, 0,578; 0,700)
	A_8	F	F	F	P	F	F	F	P	F	(0,278, 0,456, 0,633; 0,700)
	A_9	G	G	VG	G	F	G	G	F	F	(0,467, 0,656, 0,844; 0,800)
	A_{10}	G	VG	G	G	VG	G	VG	VG	G	(0,633, 0,789, 0,944; 0,900)
	A_{11}	G	F	G	VG	G	G	G	G	G	(0,511, 0,700, 0,889; 0,800)
	A_{12}	VP	P	VP	F	P	F	F	F	P	(0,222, 0,367, 0,511; 0,600)

Table 3d. Average~~d~~ ratings of suppliers versus the willingness criteria

**Criterion~~a~~**	**Supplier~~s~~**	**Decision makers**									**Aggregated ratings r_{ij}**
		t_1			t_2			t_3			
		D_1	D_2	D_3	D_1	D_2	D_3	D_1	D_2	D_3	
W_1	A_1	P	F	P	F	F	P	G	F	G	(0,311, 0,478, 0,644; 0,700)
	A_2	G	VG	VG	G	G	G	G	G	VG	(0,600, 0,767, 0,933; 0,900)
	A_3	G	F	G	VG	G	G	VG	G	G	(0,544, 0,722, 0,900; 0,800)
	A_4	VG	G	G	G	VG	G	G	G	G	(0,567, 0,744, 0,922; 0,900)
	A_5	G	F	G	G	G	G	F	F	G	(0,433, 0,633, 0,833; 0,800)
	A_6	G	VG	G	G	G	G	VG	G	G	(0,567, 0,744, 0,922; 0,900)
	A_7	G	G	G	F	G	G	G	F	G	(0,456, 0,656, 0,856; 0,800)
	A_8	P	F	F	P	F	P	F	G	F	(0,289, 0,456, 0,622; 0,700)
	A_9	G	G	VG	G	F	G	F	G	G	(0,489, 0,678, 0,867; 0,800)
	A_{10}	F	P	F	G	F	G	G	F	F	(0,356, 0,544, 0,733; 0,700)
	A_{11}	F	G	F	G	G	F	G	F	G	(0,411, 0,611, 0,811;

											0,800)
	A_{12}	P	VP	P	P	P	F	G	VG	G	(0,333, 0,467, 0,600; 0,600)
W_2	A_1	F	P	F	F	P	F	F	P	F	(0,267, 0,433, 0,600; 0,700)
	A_2	G	G	VG	F	G	F	G	G	VG	(0,522, 0,700, 0,878; 0,800)
	A_3	G	F	G	G	G	G	G	G	G	(0,478, 0,678, 0,878; 0,800)
	A_4	F	G	G	G	F	G	G	F	G	(0,433, 0,633, 0,833; 0,800)
	A_5	F	G	G	G	VG	G	VG	G	G	(0,544, 0,722, 0,900; 0,800)
	A_6	F	F	G	G	G	VG	G	F	G	(0,467, 0,656, 0,844; 0,800)
	A_7	G	G	VG	G	G	G	G	G	G	(0,533, 0,722, 0,911; 0,900)
	A_8	P	VP	P	P	P	F	F	F	G	(0,256, 0,400, 0,544; 0,600)
	A_9	G	G	F	G	F	F	F	G	F	(0,389, 0,589, 0,789; 0,800)
	A_{10}	G	F	F	G	G	F	G	F	G	(0,411, 0,611, 0,811; 0,800)
	A_{11}	F	G	G	G	G	G	G	G	VG	(0,511, 0,700, 0,889; 0,800)
	A_{12}	G	G	G	G	F	G	G	F	G	(0,456, 0,656, 0,856; 0,800)

Table 3e. Average~~d~~ ratings of suppliers versus the willingness criteria

Formatted Table

**Criterion ~~a~~**	**Supplier~~s~~**	**Decision maker~~s~~**									**Aggregated ratings r_{ij}**
		t_1			t_2			t_3			
		D_1	D_2	D_3	D_1	D_2	D_3	D_1	D_2	D_3	
W_3	A_1	P	P	P	F	P	F	F	P	F	(0,244, 0,389, 0,533; 0,700)
	A_2	F	F	G	F	G	F	G	G	G	(0,411, 0,611, 0,811; 0,800)
	A_3	G	G	F	F	G	F	F	G	F	(0,389, 0,589, 0,789; 0,800)
	A_4	F	G	F	G	G	G	F	G	G	(0,433, 0,633, 0,833; 0,800)
	A_5	G	F	G	G	F	G	G	G	G	(0,456, 0,656, 0,856; 0,800)
	A_6	F	G	F	G	F	F	VG	G	VG	(0,478, 0,656, 0,833; 0,800)
	A_7	G	VG	VG	G	VG	G	G	G	G	(0,600, 0,767, 0,933; 0,900)
	A_8	P	P	P	F	P	F	F	F	G	(0,278, 0,433, 0,589; 0,700)
	A_9	G	VG	G	VG	VG	G	F	G	G	(0,578, 0,744, 0,911; 0,800)

	A_{10}	F	G	G	G	G	G	G	VG	G	(0,511, 0,700, 0,889; 0,800)
	A_{11}	G	G	G	G	VG	G	G	G	G	(0,533, 0,722, 0,911; 0,900)
	A_{12}	VG	G	G	G	G	G	G	G	VG	(0,567, 0,744, 0,922; 0,900)
W_4	A_1	F	F	F	P	F	F	G	F	G	(0,333, 0,522, 0,711; 0,700)
	A_2	G	G	G	G	F	G	F	G	F	(0,433, 0,633, 0,833; 0,800)
	A_3	F	G	F	G	G	F	G	G	F	(0,411, 0,611, 0,811; 0,800)
	A_4	G	VG	G	G	G	G	G	G	G	(0,533, 0,722, 0,911; 0,900)
	A_5	F	F	F	P	F	P	G	F	G	(0,322, 0,500, 0,678; 0,700)
	A_6	F	G	G	F	F	G	VG	VG	G	(0,500, 0,678, 0,856; 0,800)
	A_7	F	F	G	G	F	F	G	F	F	(0,367, 0,567, 0,767; 0,800)
	A_8	F	F	F	F	G	F	G	F	G	(0,367, 0,567, 0,767; 0,800)
	A_9	G	G	G	F	G	F	G	F	G	(0,433, 0,633, 0,833; 0,800)
	A_{10}	F	G	F	F	G	G	G	G	F	(0,411, 0,611, 0,811; 0,800)
	A_{11}	G	F	G	F	F	F	G	G	G	(0,411, 0,611, 0,811; 0,800)
	A_{12}	G	G	G	G	VG	G	VG	G	VG	(0,600, 0,767, 0,933; 0,900)

Response to Referees' Comments

PONE-D-20-03268

A DYNAMIC GENERALIZED FUZZY MULTI-CRITERIA GROUP DECISION MAKING APPROACH FOR GREEN SUPPLIER SEGMENTATION

The authors greatly appreciate the time and effort the referees spent on reviewing this manuscript. This paper has been revised based on the constructive comments and suggestions made by the referees. Major changes are shown in red color.

Referee 1's Comments:

I would like to thank the authors for this interesting approach in dealing with an important subject. The subject of segmentation and especially weighting using decision makers or "experts" are one of the areas of debate in many fields. I found this approach easy to follow and reproducible. This is an important advantage for the proposed method. However, I have few issues that I want to recommend and clarify:

1. I am not sure that you have presented enough discussion in the literature about the added value for your approach and the need for this approach, not only from operational point of view but also from computational one. In other words, you provided in the second paragraph of the introduction why you are proposing this approach, but there is no justification for where you think it will improve the current method statistically. Is this approach better in your opinion and why?
2. Tables 6a - 6e are a bit confusing within the manuscript, maybe including these tables in an annex will be more convenient.
3. There must be a discussion section after the section "Comparison of the proposed method with another fuzzy MCDM method" and the conclusion section. The results are not discussed clearly for the reader. The results need to be interpreted from mathematical and operational point of view, as a reader I am afraid I need more explanation for the numbers. It looked in some places that you jumped from section to another without explaining the results. Moreover, you need to discuss the strengths of your approach, how it tackled current existing problem, and why do you think it should be considered by others. For example, have you considered a simulation study and compare the results with other methods to assess the consistency of the results?! or have you considered comparing this method with more statistically based approaches such as Multidimensional Latent Class

Item Response Theory Models?! There should be more discussion before you present your conclusion.

Responses:

1. Thank you very much for your comments. The authors have added some sentences in the introduction and literature review section to discuss more about the shortcomings of the existing approaches and the advantages of our approach.
2. Thanks for your suggestion. The authors have moved Tables 6a - 6e to appendix section.
3. Thanks for your comments. The authors have added some paragraphs to discuss about the results of the study and the advantages of our approach. Some sentences have been added in the implementation section to explain more about the calculation process. In this study, a new dynamic generalized fuzzy MCDM approach has been proposed. Then, we have compared the proposed method with another fuzzy MCDM method to show its advantages. The comparison between our proposed approach with more statistically based approaches such as Multidimensional Latent Class Item Response Theory Models should be our further research.

Referee 2's Comments:

While new methods for "green" supplier segmentation is certainly important, interesting, and relevant, there are several issues in this paper.

1. The methods in this paper appear to be sound, it is very hard to read and comprehend. The organization and visualization of data/results is overall, poor.
2. Background on fuzzy numbers is lengthy and a bit hard to follow.
3. There is an excess of tables, which is incredibly overwhelming and unhelpful given the complexity of the topic and notation. The tables in the literature review section are redundant or unnecessary. If tables really are necessary, for this many tables, they belong in an appendix.
4. Some terms or abbreviations are not explained and confusing. For example, in Table 6a, I'm assuming "fa" = "fair", "Ve_go" = "Very good"? This needs to be standardized and presented in a more meaningful, insightful, and visually interesting manner. For example, map responses to numbers rather than letter abbreviations, and plot a heat map of responses, rather than use a table. This can be done with ALL of the tables in this section.

5. Table 8 may be better off as some sort of visual representation (chart) rather than a table.
6. There are grammatical mistakes throughout the paper

Responses:

1. Thank you very much for your comments. The authors have added some sentences in the implementation section to explain more about the data and results of this study. The authors have also moved the Tables 6a - 6e to the appendix.
2. Thanks for your suggestion. The authors have moved the background on fuzzy numbers to appendix.
3. Thanks for your suggestion. The authors have moved Tables 1-3 to the appendix.
4. Thanks for your suggestion. The authors have tried to change the abbreviations of linguistic variables (Appendix B - Table 2 and other tables).
5. Thanks for your suggestion. The authors have modified the Table 8 to make it more visually.
6. The authors have tried to fix the grammatical mistakes throughout the paper.

The authors would like to thank again the reviewers for the time and expertise they have invested in these reviews. The revised manuscript with marked changes has been resubmitted to your journal. We look forward to your positive response.

Sincerely,

Luu Quoc Dat
